# In vivo visualization and molecular targeting of the cardiac conduction system

**DOI:** 10.1172/JCI156955

**Published:** 2022-10-17

**Authors:** William R. Goodyer, Benjamin M. Beyersdorf, Lauren Duan, Nynke S. van den Berg, Sruthi Mantri, Francisco X. Galdos, Nazan Puluca, Jan W. Buikema, Soah Lee, Darren Salmi, Elise R. Robinson, Stephan Rogalla, Dillon P. Cogan, Chaitan Khosla, Eben L. Rosenthal, Sean M. Wu

**Affiliations:** 1 Cardiovascular Institute, Stanford University School of Medicine, Stanford, California, USA.; 2 Department of Pediatrics, Stanford University, Stanford, California, USA.; 3 Department of Cardiovascular Surgery, Institute Insure (Institute for Translational Cardiac Surgery), German Heart Center Munich, Technische Universität München, Munich, Germany.; 4 Department of Otolaryngology-Head and Neck Surgery, Stanford University School of Medicine, Stanford, California, USA.; 5 Department of Cardiology, Utrecht Regenerative Medicine Center, University Medical Center Utrecht, Utrecht University, Utrecht, Netherlands.; 6 Department of Cardiology, Amsterdam University Medical Center, Location VUmc, Amsterdam, Netherlands.; 7 Department of Pharmacy, Bioconvergence Program, Sungkyunkwan University, Suwon, South Korea.; 8 Department of Pathology and; 9 Department of Radiology, Stanford University, Stanford, California, USA.; 10 Division of Gastroenterology, Department of Medicine, Stanford University School of Medicine, Stanford, California, USA.; 11 Departments of Chemistry and Chemical Engineering and Sarafan ChEM-H Institute, Stanford University, Stanford, California, USA.; 12 Department of Otolaryngology-Head and Neck Surgery, Vanderbilt University Medical Center, Nashville, Tennessee, USA.; 13 Division of Cardiovascular Medicine, Department of Medicine, Stanford University School of Medicine, Stanford, California, USA.

**Keywords:** Cardiology, Arrhythmias, Cardiovascular disease, Diagnostic imaging

## Abstract

Accidental injury to the cardiac conduction system (CCS), a network of specialized cells embedded within the heart and indistinguishable from the surrounding heart muscle tissue, is a major complication in cardiac surgeries. Here, we addressed this unmet need by engineering targeted antibody-dye conjugates directed against the CCS, allowing for the visualization of the CCS in vivo following a single intravenous injection in mice. These optical imaging tools showed high sensitivity, specificity, and resolution, with no adverse effects on CCS function. Further, with the goal of creating a viable prototype for human use, we generated a fully human monoclonal Fab that similarly targets the CCS with high specificity. We demonstrate that, when conjugated to an alternative cargo, this Fab can also be used to modulate CCS biology in vivo, providing a proof of principle for targeted cardiac therapeutics. Finally, in performing differential gene expression analyses of the entire murine CCS at single-cell resolution, we uncovered and validated a suite of additional cell surface markers that can be used to molecularly target the distinct subcomponents of the CCS, each prone to distinct life-threatening arrhythmias. These findings lay the foundation for translational approaches targeting the CCS for visualization and therapy in cardiothoracic surgery, cardiac imaging, and arrhythmia management.

## Graphical abstract

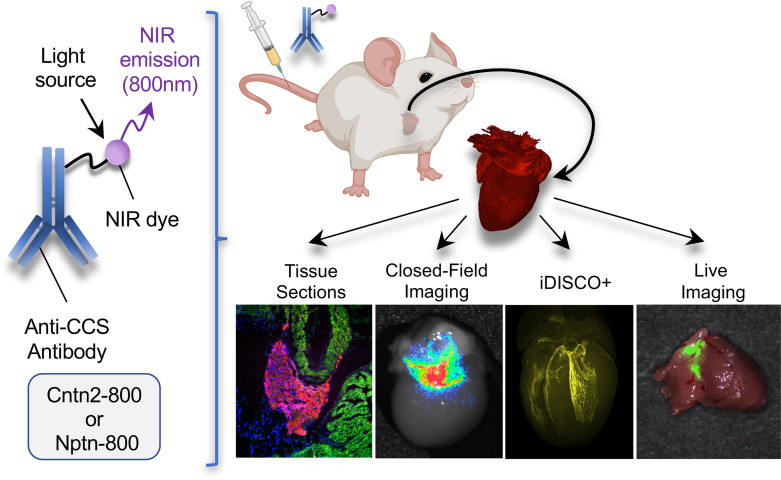

## Introduction

The cardiac conduction system (CCS) is made up of specialized heart cells that establish the rhythmic beating of the heart through coordinated contraction of its chambers (1). Damage to the CCS can result in decreased cardiac function, life-long need for electronic pacemakers, and fatal arrhythmias (2). The CCS is invisible to the naked eye and, as such, is often accidentally damaged during intracardiac surgeries. In fact, postoperative heart block, secondary to accidental surgical damage of the atrioventricular node (AVN), complicates roughly 1% to 3% of all congenital heart disease surgeries and 4% to 24% of more complex repairs (3, 4). Current standard of care remains the use of anatomical landmarks to approximate the location of the CCS. To date, there exists no commercially available method for cardiac surgeons to visualize the CCS in the operating suite.

Optical imaging using molecularly targeted antibodies conjugated to fluorescent dyes is a burgeoning technology within translational medicine, providing potential opportunities in diagnostics (e.g., tumor burden detection) and image-guided surgery in order to improve surgical outcomes (5). The vast majority of research into optical imaging to date has focused on tumor detection for intraoperative image-guided oncologic resections, with several of these agents currently being evaluated in clinical trials (6). Optical imaging agents theoretically also have incredible potential for visualizing and thus sparing normal tissues often inadvertently damaged during invasive procedures. Particular hurdles to broadening this technology to more complex structures such as the heart include (a) the lack of distinguishing surface markers, (b) cell-type heterogeneity, (c) depth below the tissue surface limiting both the delivery and visualization of the fluorescent signal, (d) complex 3D anatomy, and (e) inherent challenges in performing intracardiac imaging in the operating suite (7, 8).

To address the unmet medical need of preventing iatrogenic injury to the CCS during cardiac procedures, we leveraged the known cell surface marker contactin 2 (*Cntn2*) that is specifically expressed within the CCS (9). We generated a targeted antibody-dye conjugate, “mCntn2-800,” consisting of a near-infrared (NIR) dye (800 nm) covalently conjugated to a commercial polyclonal antibody directed against the extracellular mouse Cntn2 protein. We validated the ability of mCntn2-800 to label the entire CCS in vivo following a single intravenous injection in mice. We then generated a fully human monoclonal Fab antibody directed against human CNTN2 and validated its ability to bind and target a cytotoxin to modulate the biology of the mouse CCS in vivo. We next harnessed the power of single-cell transcriptomics to determine and validate additional unique surface markers within individual CCS subcomponents. Finally, we confirmed the generalizability of our in vivo targeting approach by generating and validating “mNptn-800,” an antibody-dye conjugate directed against murine neuroplastin (*Nptn*), a CCS marker conserved in both mice and humans. These results demonstrate, for the first time to our knowledge, a proof of principle for the in vivo optical imaging and molecular targeting of the CCS. This framework provides a foundation for generating cell surface receptor–targeting antibodies for clinical imaging and precision therapy of cells in different organs, thereby revolutionizing our current approaches to in vivo imaging and drug delivery.

## Results

### Labeling of the CCS using systemic mCntn2-800.

**Figure 1:**
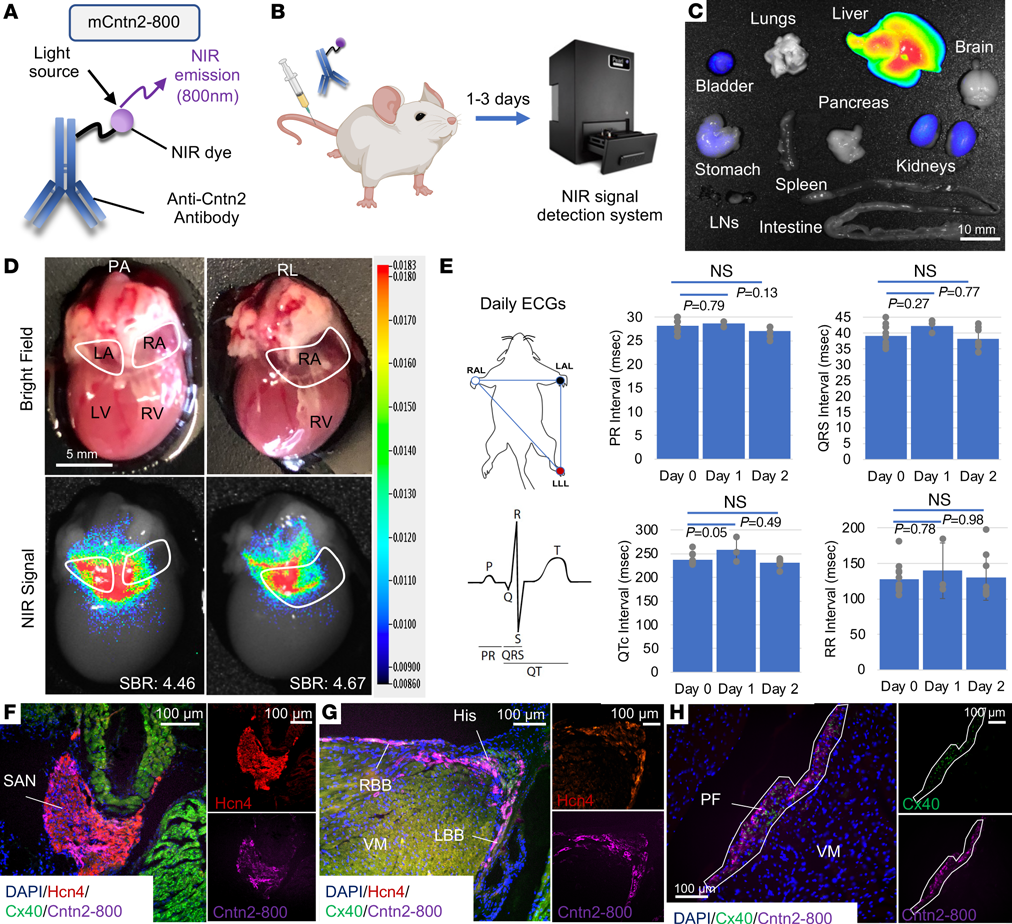
Systemic injection of mCntn2-800 in mice labels the CCS in vivo. (**A**) Antibody-dye conjugate (mCntn2-800) consists of a near-infrared (NIR) dye conjugated to an antibody against the CCS-specific surface marker Cntn2 (contactin 2). (**B**) Experimental work flow. (**C**) Whole-body biodistribution of other tissue types, showing expected clearance within the liver, bladder, and kidneys and notable absence from the brain. (**D**) Whole mouse heart (*n* = 3) from a wild-type (WT) mouse injected 3 days prior with mCntn2-800, in posterior-anterior (PA) and right lateral (RL) views. Atria are outlined in white and cardiac chambers are listed. LA, left atrium; LV, left ventricle; RA, right atrium; RV, right ventricle. Top: Brightfield. Bottom: NIR signal demonstrating labeling of the CCS (blue→red = lowest→highest signal). Mean signal to background ratio (SBR) is indicated. (**E**) Measured intervals (in ms) from sedated surface electrocardiograms (ECGs) including PR, QRS, QTc, and RR in WT mice prior to (day 0 = baseline, *n* = 12) and daily (day 1 *n* = 3, day 2 *n* = 9; after injection) following a single tail vein injection of mCntn2-800. Intervals (mean ± SD) on a given day after injection were compared to each mouse’s preinjection control baseline (day 0) using 1-way ANOVA with Tukey’s post hoc test. (**F**–**H**) Heart sections from a WT mouse injected 2 days prior with mCntn2-800. CCS components labeled with mCntn2-800 (purple) and costained with antibodies targeting known markers of the CCS, including anti-Hcn4 (red, SAN and His) or anti-Cx40 (green, PF). DAPI (blue, nuclei). LBB/RBB, left and right bundle branches; His, His bundle; PF, Purkinje fibers; SAN, sinoatrial node; VM, ventricular myocardium. Scale bars: 10 mm (**C**), 5 mm (**D**), and 100 μm (**F**–**H**).

To assess the feasibility of labeling the CCS in vivo, we conjugated IRDye800CW NHS ester, an NIR dye already in clinical use (10), to a commercially available polyclonal antibody directed against Cntn2, an extracellular marker previously shown to be expressed specifically within the CCS of mice and humans (Figure 1A) (9, 11). We injected wild-type adult mice intravenously with a single dose (75 μg) of either mCntn2-800 or control IgG-800 (i.e., nonspecific IgG conjugated to IRDye800CW NHS ester), harvested the hearts and all other major organs after 72 hours, and imaged them using a closed-field NIR imaging system (Figure 1B). NIR signal was detected expectedly within the liver and kidneys, similar to prior reports of metabolism and clearance of other NIR imaging agents (12). Notably, mCntn2-800 signal was not detected within the brain tissue despite it being the only other major organ besides the CCS known to express *Cntn2* (13), consistent with an intact blood-brain barrier (Figure 1C).

Within the heart, mCntn2-800 showed high intensity signal localized to the CCS (signal to background ratio, SBR: 4.463, SD: 0.388) as compared with mice injected with control IgG-800 (SBR: 1.810, SD: 0.177; *P =* 0.0001) (Figure 1D and Supplemental Table 1; supplemental material available online with this article; https://doi.org/10.1172/JCI156955DS1). A dose-finding study revealed that specific imaging signals were present at all doses given, with an optimal SBR at 75 μg (~2 mg/kg) but with significant signal present even at the lowest dose of 25 μg (~0.7 mg/kg) (SBR: 2.932, SD: 0.319; *P =* 0.0018) (Supplemental Figure 1, A and B, and Supplemental Table 1). A clearance study revealed that a single 75 μg dose of mCntn2-800 provides specific and intense signal even out to 4 days after the initial injection (Supplemental Figure 1, C and D, and Supplemental Table 1).

To assess for any potential toxicity of mCntn2-800 to the CCS, a surface electrocardiogram (ECG) study was performed on all mice prior to and daily for 2 days following a single 75 μg injection of mCntn2-800. There were no adverse effects of mCntn2-800 on normal CCS function, with no statistically significant changes in any measured surface ECG parameters (including PR, QRS, QTc, and RR), as compared to the mice prior to injection (Figure 1E and Supplemental Table 2). Finally, we examined the specificity of mCntn2-800 to target the CCS and confirmed the absence of its binding to non-CCS cells by serial sections and coimmunostaining with established markers of the CCS, including hyperpolarization-activated cyclic nucleotide–gated potassium channel 4 (Hcn4) and connexin 40 (Cx40) (Figure 1, F–H).

**Figure 2:**
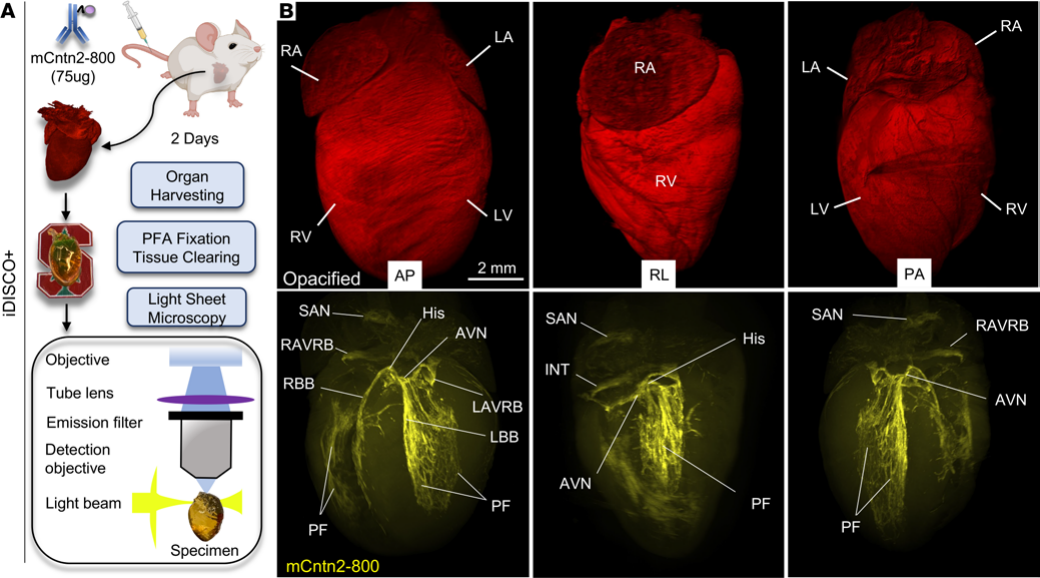
Optical clearing and 3D volumetric analyses on an intact heart following mCntn2-800 systemic injection reveals high-resolution labeling of the entire CCS. (**A**) Schematic representation of workflow for iDISCO+ clearing of mouse hearts and visualization using light-sheet microscopy. (**B**) iDISCO+ cleared heart harvested from a wild-type (CD1) mouse injected 2 days prior with mCntn2-800 (75 μg). Representative heart (*n* = 3) shown from 3 different angles of view: anterior-posterior (AP), right lateral (RL), and posterior-anterior (PA). Top and bottom rows are the same optically cleared heart using iDISCO+ where, in the top row, background fluorescence has been saturated to provide a representation of the opacified heart. Bottom row demonstrates the same tissue-cleared heart, showing near-infrared (800 nm) signal from mCntn2-800 marking the entire CCS. Conduction system components are labeled as indicated. Scale bar: 2 mm. AVN, atrioventricular node; His, His bundle; INT, internodal tracks; LA/RA, left or right atrium; LAVRB, left AV ring bundle; LBB/RBB, left or right bundle branch; LV/RV, left or right ventricle; PF, Purkinje fibers; RAVRB, right AV ring bundle; SAN, sinoatrial node.

Given the challenges of visualizing the complex 3D anatomy of the CCS with only 2D serial tissue sections, we leveraged whole-mount immunostaining and 3D imaging using tissue clearing (iDISCO+) (14) and light-sheet microscopy with volume rendering on intact, wild-type mouse hearts (Figure 2A). Specifically, following a single 75 μg injection of mCntn2-800, whole hearts were harvested after 48 hours, fixed, and cleared using iDISCO+. Consistent with 2D immunofluorescence sections, the cleared hearts showed exquisite specificity and high resolution of mCntn2-800 signal throughout the entire CCS (Figure 2B and Supplemental Video 1).

### Live imaging of the CCS following a single intravenous injection of mCntn2-800.

**Figure 3:**
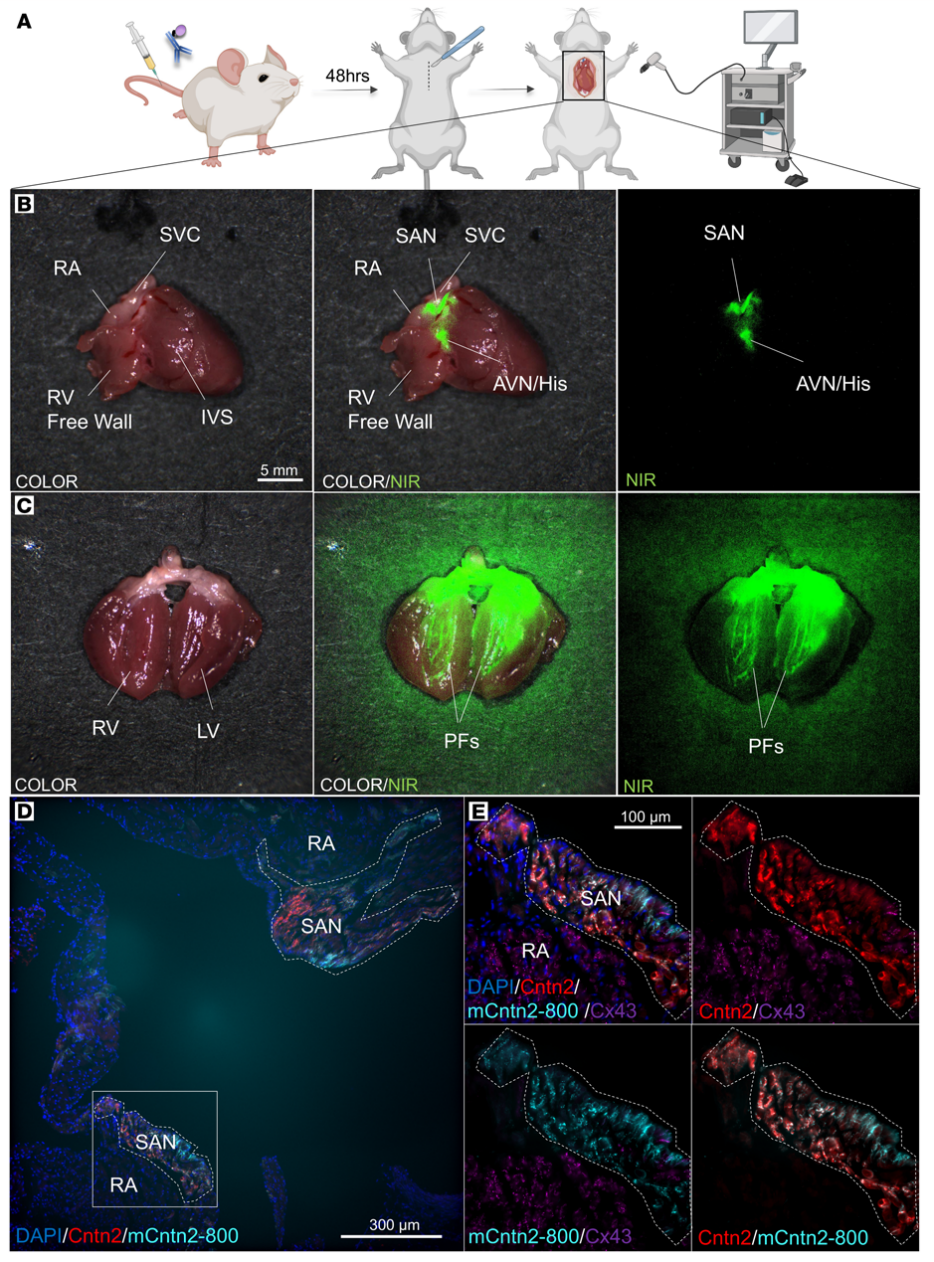
Live imaging of the murine CCS following mCntn2-800 systemic injection. (**A**) Experimental workflow: wild-type mice (*n* = 3) received a tail vein injection of mCntn2-800 and after 48 hours were sedated and received a sternotomy and cardiac incisions including a right atriotomy and right ventriculotomy to simulate a surgical scenario. Live imaging of the heart with a FLARE Intraoperative Near-Infrared (NIR) Fluorescence Imaging System. (**B** and **C**) Heart with visible sinoatrial node (SAN), atrioventricular node/His bundle (AVN/His), and Purkinje fiber (PF) network. Left: Color image of ex vivo heart. Right: mCntn2-800 NIR signal (green). Middle: Merged image of color image and NIR (green) signal. (**D**) Heart sections from the same heart, demonstrating mCntn2-800 signal (cyan) labeling the SAN, as costained with anti-Cntn2 (red) immunostaining. (**E**) Magnified region in SAN indicated by the white box in **D**. Right atrial (RA) tissue labeled with anti-Cx43 (purple). DAPI (blue, nuclei). IVS, interventricular septum; RV, right ventricle; SAN, sinoatrial node; SVC, superior vena cava. Scale bars: 5 mm (**B** and **C**), 300 μm (**D**), and 100 μm (**E**).

Next, we sought to perform live imaging of the CCS using a FLARE Intraoperative NIR Fluorescence Imaging System previously used in clinical trials for tumor detection (15). Wild-type mice received a tail vein injection of mCntn2-800 and after 48 hours were sedated and received a sternotomy followed by a right atriotomy and ventriculotomy to expose the endocardial surface of the heart, analogous to an intracardiac surgery (Figure 3A). In the still fibrillating heart, a comma-shaped NIR^+^ structure was visualized at the border of the superior vena cava (SVC) and right atrial (RA) junction, consistent with labeling of the sinoatrial node (SAN) (Figure 3B and Supplemental Videos 2 and 3). Additionally, a nodal structure located at the crux of the heart was appreciated consistent with the AVN and contiguous His bundle diving anteriorly into the interventricular septum (Figure 3B and Supplemental Videos 2 and 3) as well as the reticular Purkinje fiber (PF) network (Figure 3C and Supplemental Video 4). Notably, the NIR signal could be seen moving in time with manipulation of the live, fibrillating cardiac tissue. The specificity of the NIR^+^ structures was subsequently confirmed by fixation and immunostaining of the whole heart. NIR signal specifically labeled all CCS components and was notably absent from other major cardiac cell types, including the surrounding Cx43-positive working myocardium and CD31-positive vascular endothelium (Figure 3, D and E, and Supplemental Figure 2).

### Generation and characterization of a fully human anti-CNTN2 Fab antibody that targets the CCS in vivo.

To facilitate clinical translation and with support from a commercial vendor, we employed a phage display strategy to screen for a fully human monoclonal antibody Fab that binds with high affinity to the human recombinant CNTN2. From initial hits, we validated our lead Fab to bind human recombinant CNTN2 in vitro (Supplemental Figure 3, A and B) and subsequently generated a Fab-IRDye800CW conjugate (hCNTN2-800) by covalent modification with the IRDye800CW NHS ester (Supplemental Figure 3C). hCNTN2-800 was then introduced into wild-type mice by a single tail vein injection. After 1 day, their hearts were harvested, fixed, and sectioned for immunofluorescence staining (Supplemental Figure 3D). Immunofluorescence costaining for Cx40, a well-known marker of PF cells, demonstrates that hCNTN2-800 can successfully target the CCS in vivo (Supplemental Figure 3E).

**Figure 4:**
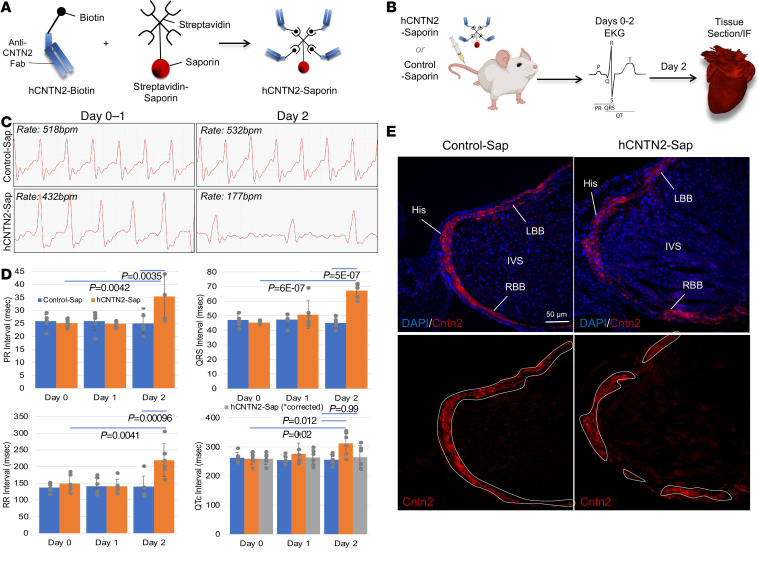
Anti-CNTN2 Fab successfully targets alternative cargo to the CCS. (**A**) Human anti-CNTN2 Fab antibody was biotinylated and conjugated to streptavidin-linked saporin, a cell toxin (hCNTN2-Sap). (**B**) Wild-type mice received a single tail vein injection of either hCNTN2-Sap (100 μg) (*n* = 6) or Control-Sap (100 μg nonspecific human IgG similarly conjugated to saporin) (*n* = 6). Mice received electrocardiograms (ECGs) on day 0 (baseline) and daily following injection with control or hCNTN2-Sap. On day 2, hearts were harvested, fixed, and immunostained. (**C**) Representative ECG tracings (*n* = 6 per condition). (**D**) By day 2, mice injected with hCNTN2-Sap demonstrated marked conduction abnormalities, including prolonged PR, QRS, and RR intervals as compared with mice injected with Control-Sap (mean intervals in ms ± SD). Gray bar, QTc interval corrected for QRS intervals. Statistical analyses using 2-way ANOVA with Tukey’s post hoc test. (**E**) Consistent with targeted cell death, immunofluorescence of the CCS (red) showed subtotal loss of CCS cells as shown within the His bundle (His), right and left bundle branches (RBB/LBB) as stained by anti-Cntn2. His, His bundle; IVS, interventricular septum; LBB, left bundle branch; RBB, right bundle branch. Scale bar: 50 μm.

Beyond their use in in vivo imaging of the CCS, the availability of a CCS-targeting antibody may also facilitate the treatment of arrhythmias by precision delivery of drugs or alternative cargos capable of modulating CCS cell function. To examine the ability of our monoclonal Fab to target and functionally perturb the CCS, we biotinylated the hCNTN2 Fab and conjugated it to streptavidin bound to saporin, a cellular toxin that is incapable of receptor-mediated internalization through cell surface membranes. The incubation of these 2 products resulted in “hCNTN2-Sap” (Figure 4A). We then injected wild-type mice with a single tail vein injection of either hCNTN2-Sap (100 μg, *n =* 6) or Control-Sap (100 μg of nonspecific biotinylated human IgG conjugated to streptavidin-saporin, *n =* 6) and harvested hearts 2 days later (Figure 4B). These mice received baseline (day 0) and daily ECGs following injection to assess for conduction system disruption. On day 2 after injection, mice injected with hCNTN2-Sap consistently showed markedly abnormal cardiac rhythm, including prolonged RR (219 ms vs. 140 ms in Controls; *P =* 0.0001), PR (35 ms vs. 25 ms in Controls; *P =* 0.0035), and QRS (67 ms vs. 45 ms in Controls; *P =* 5 × 10^–7^) intervals (Figure 4, C and D, and Supplemental Table 3), consistent with abnormal function within the SAN, AVN, and His bundle branches/PFs, respectively. While QTc intervals, reflective of ventricular myocardium repolarization, were also prolonged (312 ms vs. 256 ms in Controls; *P =* 0.012), when corrected for the widened QRS intervals, no significant difference was noted (265 ms vs. 256 ms in Controls; *P =* 0.99), again consistent with conduction-cell-specific targeting within the heart. We then confirmed these ECG findings with immunohistochemical analyses of the CCS structure and found that the CCS of hCNTN2-Sap–injected mice showed widespread loss of cardiac conduction cells in all major CCS components, consistent with their targeted cell death (Figure 4E and Supplemental Figure 4). Finally, consistent with the lack of signal within the central nervous system, immunostaining of hCNTN2-Sap–injected mice showed no evidence of increased neuronal cell death in the brain as compared to controls (Supplemental Figure 5).

### Gene expression analyses of murine CCS single-cell RNA sequencing data set reveals a suite of cell-surface-protein genes enriched within the CCS.

Given our successes thus far with antibody targeting of Cntn2 for in vivo imaging and phenotypic modulation of CCS cells, we next sought to identify additional cell surface markers within the CCS for substructure-specific targeting. To do this, we leveraged our previously validated single-cell RNA sequencing (scRNA-seq) data set of the entire developing murine CCS (16). Specifically, we screened for all significantly enriched genes within single cells from each major subcomponent of the CCS, including the SAN, AVN, His bundle (His), and PF cells as compared to all other cells within the heart. We prioritized our genes of interest by (a) log fold enrichment within the CCS as compared to other cell types, (b) significance (adjusted *P* value based on Bonferroni’s correction), and (c) their inclusion using the recently published SurfaceGenie algorithm (17), a web-based application for high-throughput candidate surface marker prioritization. All putative cell-surface-protein genes were then confirmed by manual assessment of their proteins’ subcellular localization using the UniProt Knowledgebase (18). These analyses resulted in the discovery of a number of CCS-enriched cell-surface-protein genes, both known (e.g., SAN *Hcn4*: 0.599 average log FC, adjusted *P* = 1.29 × 10^–247^; AVN/His *Cav3*: 0.444 average log FC, adjusted *P* = 3.51 × 10^–51^; PF *Gja5*: 0.972 average log FC, adjusted *P* = 1.17 × 10^–183^) (19, 20) and potentially novel within the CCS (Supplemental Table 4).

**Figure 5:**
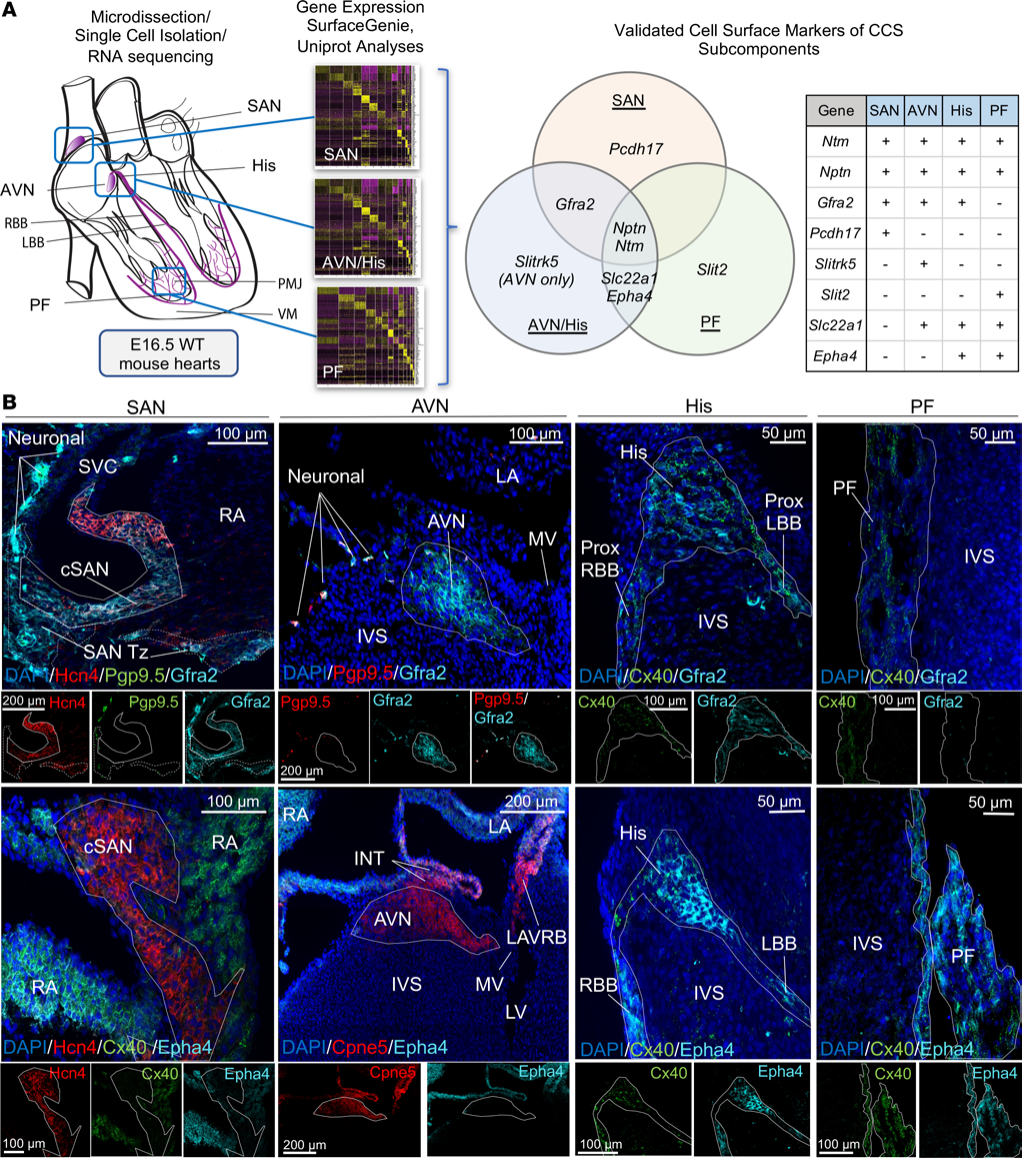
scRNA-seq analyses reveal cell surface markers within distinct components of the CCS for generating targeted optical imaging tools. (**A**) Workflow of single-cell RNA sequencing (scRNAseq) analyses to uncover cell-surface-protein genes enriched within the murine CCS subcomponents, including the sinoatrial node (SAN), atrioventricular node (AVN), His bundle (His), and Purkinje fiber (PF) cells, as compared with all other cardiac cell types. (**B**) Immunofluorescence (IF) staining of wild-type murine, embryonic day 16.5 cardiac tissue sections (*n* = 3 per marker). Distinct CCS components shown, including the SAN, AVN, His, bundle branches (BB), and PF cells (each component outlined by a solid line) for 2 validated gene markers, *Gfra2* and *Epha4*. DAPI (blue) in all images. Top panel: IF with staining against Gfra2 protein (cyan) and known markers Hcn4 (SAN, red), Cx40 (His, BB and PF, green), and Pgp9.5 (neurons, green or red as indicated). Transitional cells demarcated by hashed lines. Bottom panel: IF with staining against Epha4 protein (cyan) and known markers Hcn4 (SAN, red), Cpne5 (AVN, red), and Cx40 (His, BB and PF, green). cSAN, compact SAN; INT, internodal tract; IVS, interventricular septum; MV, mitral valve; LA, left atrial myocardium; LBB, left bundle branch; LV, left ventricle; PMJ, Purkinje-myocyte junction; Prox, proximal; RA, right atrial myocardium; RBB, right bundle branch; SAN Tz, SA nodal transitional cells; VM, ventricular myocardium. Scale bars: 100 μm (SAN, AVN [top]), 200 μm (AVN [bottom]), and 50 μm (His, PF).

From this larger list of putative CCS genes encoding cell surface proteins, we identified those enriched within each distinct CCS component including the SAN (*Pcdh17*: 0.632 average log FC, adjusted *P* = 6.01 × 10^–53^), AVN (*Slitrk5*: 0.273 average log FC, adjusted *P* = 2.16 × 10^–60^), and PF cells (*Slit2*: 0.695 average log FC, adjusted *P* = 2.57 × 10^–62^) (Figure 5A and Supplemental Table 5). Additional cell-surface-protein genes were found to be enriched in distinct combinations of subcomponents, including the nodal tissue (SAN/AVN/His, *Gfra2*), ventricular conduction system (AVN/His/PF, *Slc22a1*), distal fast-conduction CCS (His/PF, *Epha4*), as well as throughout the entire CCS (neurotrimin [*Ntm*] and neuroplastin [*Nptn*]) (Figure 5A and Supplemental Table 5). While *Nptn* was not initially found to be significantly enriched within AVN/His cells when compared to all other cell types, it was indeed significantly enriched when compared specifically with all other cardiomyocyte clusters (AVN/His *Nptn*: 0.303 average log FC, adjusted *P* = 4.69 × 10^–22^) (data not shown).

### Validation of cell surface markers within distinct components of the murine CCS.

Consistent with our bioinformatics approach, one of the most enriched cell-surface-marker genes uncovered in our analyses was *Ntm*, encoding a member of the IgLON (LAMP, OBCAM, Ntm) family of immunoglobulin (Ig) domain–containing GPI-anchored cell adhesion molecules, that we have previously validated to be specifically expressed throughout the entire CCS (16). In order to validate the other 7 cell surface candidates, we next employed high-resolution fluorescence in situ hybridization (RNAscope) or immunostaining analyses of wild-type murine heart sections. The cell type expression pattern of each gene candidate predicted from our scRNA-seq expression data (Figure 5A) was fully recapitulated by tissue staining in wild-type mouse hearts with high specificity, including *Pcdh17* (SAN), *Slitrk5* (AVN), *Slit2* (PF), *Gfra2* (SAN/AVN/His), *Slc22a1* (AVN/His/BB/PF), and *Epha4* (His/BB/PF) (Figure 5B and Supplemental Figures 6–9). *Gfra2* was additionally noted to be expressed in a rare cell subpopulation surrounding the SAN that were positive for the pan-neuronal marker protein gene product 9.5 (Pgp9.5) (Figures 5B), consistent with a cardiac neuronal fate (21). Additionally, *Gfra2* was expressed in a subset of vimentin-positive cells surrounding the large penetrating vessels of the ventricles (Supplemental Figure 7).

**Figure 6:**
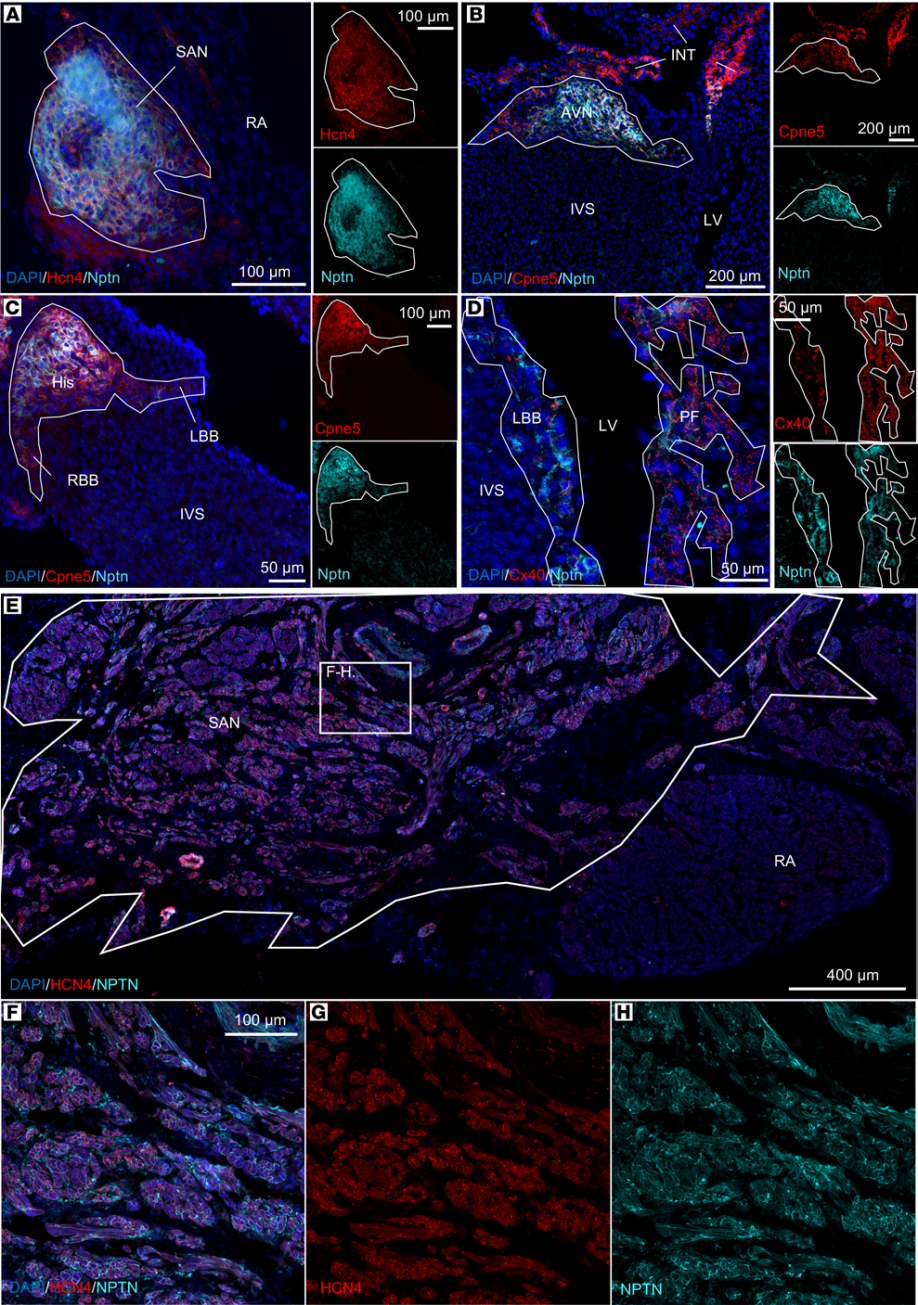
Nptn is enriched throughout the murine and human CCS. Immunofluorescence staining of wild-type, postnatal day 10 mouse (**A**–**D**) (*n* = 3) and 65-year-old human (**E**–**H**) (*n* = 3) cardiac tissue sections. (**A**–**D**) Mouse CCS: anti-Nptn protein staining (cyan) within the (**A**) sinoatrial node (SAN) marked by *Hcn4* (red); (**B**) atrioventricular node (AVN) labeled by *Cpne5* (red); (**C**) His bundle (His), right bundle branch (RBB) and left bundle branch (LBB) indicated by *Cpne5* (red); and (**D**) Purkinje fibers marked by *Cx40* (red). DAPI (blue) in all panels. (**E**–**H**) Human CCS: Anti-NPTN (cyan) labeling the SAN, costained for HCN4 (red). Magnified region in SAN (**F** and **H**) indicated by white box in **E**. DAPI (blue). INT, internodal tracts; IVS, interventricular septum; LV, left ventricle; RA, right atrial myocardium. Scale bars: 100 μm (**A**, right images in **C**, and **F**), 200 μm (**B**), 50 μm (left image in **C** and **D**), and 400 μm (**E**).

To validate the in vivo targeting specificity of a CCS marker identified from our scRNA-seq data set, we focused on the expression of neuroplastin (*Nptn*), a type I transmembrane protein belonging to the Ig superfamily expressed within the central nervous system (22) in the murine and human heart (Figure 6). Nptn was found histologically to be enriched throughout the entire CCS in both adult mice (Figure 6, A–D, and Supplemental Figure 10) and adult human (Figure 6, E–H, and Supplemental Figure 11) heart tissue sections.

### In vivo labeling of the murine CCS by targeting Nptn.

**Figure 7:**
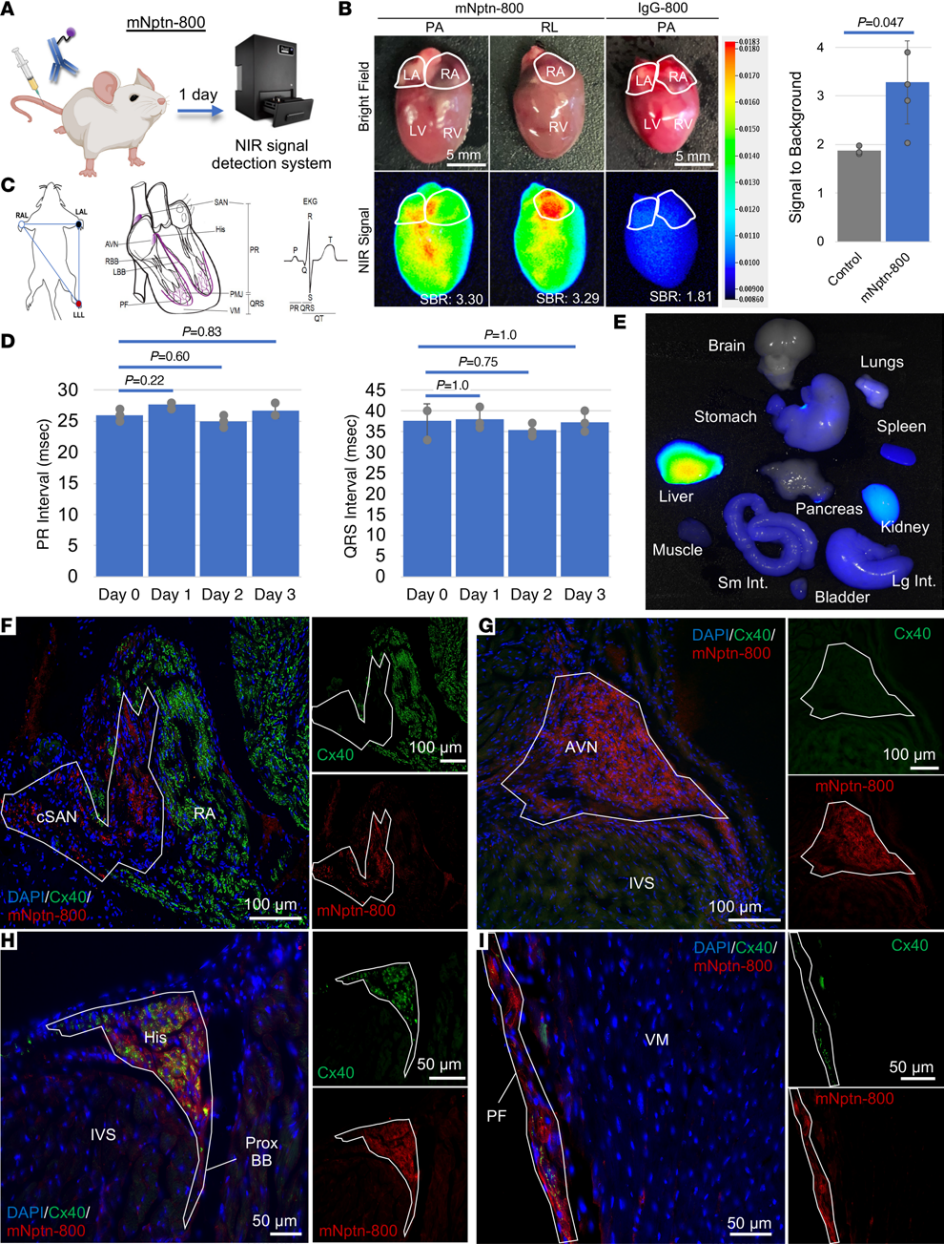
Systemic injection of mNptn-800 in mice safely labels the CCS in vivo. (**A**) Experimental work flow. (**B**) Whole hearts from a wild-type (WT) mouse injected 1 day prior with mNptn-800 (*n* = 3) or IgG-800 (*n* = 3). Heart shown in posterior-anterior (PA) and right lateral (RL) views. Atria are outlined in white and cardiac chambers are listed. LA, left atrium; LV, left ventricle; RA, right atrium; RV, right ventricle. Top: Brightfield. Bottom: Near infrared (NIR) signal demonstrating labeling of the CCS (blue→red = lowest→highest signal). Mean signal to background ratio (SBR) is indicated. Bar graph showing mean SBR in hearts exposed to 150 μg of either mNptn-800 or IgG-800 (control) (*n* = 3 each). Mean ± SD shown. Statistical analyses using 2-tailed, unpaired Student’s *t* test. (**C** and **D**) Sedated surface electrocardiograms (ECGs) with measured intervals (in ms) including PR and QRS in WT mice prior to (day 0 = baseline) and daily (day 1, day 2, day 3 = after injection) following a single tail vein injection of mNptn-800 (150 μg). *n* = 3 for all time points. Intervals on a given day after injection were compared to each mouse’s preinjection control baseline (day 0) using 1-way ANOVA with Tukey’s post hoc test. (**E**) Whole-body biodistribution of other tissue types, showing expected clearance within the liver and kidneys. (**F**–**I**) Heart sections from adult mice (*n* = 3) injected 1 day prior with mNptn-800. (**F**) Compact SAN (cSAN) labeled by mNptn-800 signal (red) and absence of Cx40 (green) as opposed to Cx40^+^ right atrial myocardium (RA). (**G**) AVN labeled with mNptn-800 (red) and consistently lacking Cx40 (green) expression. (**H**) His bundle (His) and proximal bundle branch (Prox BB) and (**I**) Purkinje fibers (PF) costained for Cx40 (green) and mNptn-800 signal (red). mNptn-800 signal was amplified using an anti-sheep 555 nm secondary antibody following tissue fixation. DAPI (blue, nuclei). IVS, interventricular septum; VM, ventricular myocardium. Scale bars: 5 mm (**B**), 100 μm (**F** and **G**), and 50 μm (**H** and **I**).

To confirm the ability of anti-Nptn antibodies to specifically target the CCS in vivo, we engineered mNptn-800 by conjugating a commercial polyclonal mouse anti-Nptn antibody to IRDye800CW NHS ester (10). Following a single intravenous tail vein injection of 150 μg of either mNptn-800 or control IgG-800 (nonspecific IgG conjugated to IRDye800CW NHS ester) in wild-type adult mice, we isolated whole hearts after 24 hours and imaged using a closed NIR camera system (Figure 7A). We found that while IgG-800 showed no specific signal within the hearts of injected mice (SBR: 1.847, SD: 0.0.086), a strong and CCS-specific signal was detected in mNptn-800–injected hearts (SBR: 3.284, SD: 0.856, *P =* 0.047) (Figure 7B and Supplemental Table 1).

To assess for potential toxicities of mNptn-800 in CCS function, all mice received preinjection baseline (day 0) and daily ECGs for up to 3 days following injection (Figure 7, C and D, Supplemental Figure 12, and Supplemental Table 6). Reassuringly, all measured ECG intervals (including PR, QRS, QTc, and RR) remained unchanged following systemic injection of mNptn-800 as compared to IgG-800, consistent with a lack of toxicity to the CCS by mNptn-800.

We next assessed the biodistribution of mNptn-800 in other organs using closed-field NIR imaging. Following systemic delivery of the optical imaging agent, organs were freshly harvested and signal was detected expectedly within the liver and kidneys, similar to mCntn2-800 and clearance of other optical imaging agents in these organs (Figure 7E) (12). No signal was detected within the brain tissue despite the known expression of *Nptn* within the central nervous system (22), again consistent with exclusion by an intact blood-brain barrier. Finally, to assess signal specificity of mNptn-800 at the cellular level, serial heart sections of systemically injected mice were costained with known protein markers of the CCS. mNptn-800 signal was localized specifically to all components of the CCS but not to surrounding heart muscle tissue (Figure 7, F–I).

## Discussion

During cardiothoracic surgery, iatrogenic damage to the CCS, which surrounds many key interventional targets (e.g., heart valves), can result in decreased heart function and a host of irreversible, life-threatening arrhythmias (e.g., heart block), often requiring permanent pacemaker device placement (23). To address this unmet medical need, we generated a systemically injected, targeted molecular imaging tool (mCntn2-800) that allowed for live visualization of the CCS in murine hearts with high sensitivity, specificity, and resolution. mCntn2-800 represents, to our knowledge, the first ever method for the in vivo targeting of any cardiac substructure. Further, with the goal of translating our basic findings to a viable prototype for human use, we generated a fully human monoclonal Fab, directed against human CNTN2 protein, that similarly targets the CCS with high specificity and is able to target CCS cells with other cargo that can modulate CCS cell biology. Finally, in performing differential gene expression analyses of the entire murine CCS at single-cell resolution, we uncovered and validated a suite of additional cell surface markers that can potentially be used to molecularly target the distinct subcomponents of the CCS.

Current standard of care in cardiac surgery remains the use of gross anatomical landmarks to approximate the location of the CCS, otherwise indistinguishable from the surrounding heart muscle tissue. Over the past several decades, attempts have been made toward the detection of the CCS during intracardiac surgeries with the goal of preventing iatrogenic surgical damage. Earlier efforts dating back to the 1960s included measuring the impedance or ECGs through the direct placement of electrodes on the heart tissue (24). These approaches, however, have been hampered by inadequate resolution, variable detection based on heterogeneity in CCS tissue depth, and/or the need for ongoing electrical activity (i.e., not viable for surgeries requiring cardioplegia). More recent efforts in the past several years have employed fiberoptic confocal microscopy (FCM) coupled with a passive fluorescent dye applied directly to the tissue surface (25). Using a hand-held FCM probe, the tissue surface can be evaluated for differences in tissue microstructure suggestive of conduction versus working myocardium.

Our antibody-based optical imaging approach provides several advantages, including (a) high specificity and spatial resolution, (b) lack of additional technical expertise needed to visualize the CCS, and (c) in vivo visualization without disruption of the surgical field or workflow. Further, numerous features inherent to our antibody-based diagnostic tools help to promote viable clinical translation, including (a) extensive research into the use of antibody-based therapeutics and diagnostics in humans, resulting in well-established pharmacokinetics and safety profiles (6, 26); (b) use of an NIR dye suitable for human use and with deep signal penetration (up to ~1 cm below the tissue surface) (7, 8), sufficient for even the AVN, the deepest of the conduction structures (27, 28); and (c) preexisting high-resolution intraoperative NIR signal detection devices such as the FLARE, already in use in clinical trials (15). Integration of optical imaging diagnostic tools into the surgical management of cardiothoracic surgery has the potential to dramatically improve adverse outcomes in both pediatric and adult cardiac surgeries. Specifically, we envision that detection of the CCS through direct, intraoperative visualization by surgeons will minimize the risk of iatrogenic damage, thereby reducing hospital costs and length of stay as well as the need for lifelong pacemaker dependency and overall morbidity and mortality associated with cardiac surgeries.

Importantly, our approach additionally lends itself to the broader potential of molecular targeting of the CCS in vivo, including systemic delivery of other forms of cargos such as contrast agents or therapeutics. Use of advanced imaging, including cardiac MRI and CT, has become a critical aspect of preprocedural planning in invasive cardiac interventions (29). However, despite substantial advances in these imaging modalities, they still cannot visualize the CCS within the heart, limiting their potential use. The lack of CCS visibility coupled with the large amount of anatomical variation, in particular in children with congenital heart disease (e.g., congenitally corrected transposition of the great arteries) (30), results in persistently elevated conduction-related complication rates in cardiac procedures (4). Antibody-contrast conjugates, through the conjugation of our lead Fab with, for instance, either superparamagnetic iron oxide nanoparticles (for MRI) or gold nanoparticles (for CT), have the potential to improve current cardiac imaging modalities by unveiling, for the first time ever, the previously evasive location and 3D course of the human CCS in structurally normal and abnormal hearts using otherwise standard preprocedural imaging.

Beyond dyes and contrast agents, our antibody-based delivery method also has therapeutic potential through the targeting of alternative cargos. Here we demonstrate CCS-specific targeted ablation following a single intravenous injection of our antibody-saporin conjugate. Currently, targeted ablation of the conduction system for rate control in refractory atrial fibrillation is highly effective; however, it still necessitates an invasive electrophysiology ablation procedure (31). These proof-of-principle experiments also highlight the potential for targeting other forms of cargo for precision therapy, namely antiarrhythmic drugs. Two of the most effective intravenous antiarrhythmics available are amiodarone and procainamide. However, both are associated with unwanted systemic side effects, with direct toxicities on multiple other organ systems at standard dosing, often limiting their broader use (32). In fact, amiodarone represents approximately 30% of the world’s antiarrhythmic drug market (33); however, nearly one-third of all patients cannot tolerate long-term use of the drug due to its extracardiac adverse reactions that affect nearly every organ in the body, including the thyroid, lungs, liver, eyes, skin, central and peripheral nervous system, among others. Direct targeting of antiarrhythmics such as amiodarone to the CCS has the potential to improve treatment of life-threatening cardiac arrhythmias (e.g., junctional ectopic tachycardia) while at the same time mitigating their serious off-target effects (32). Finally, as the CCS is composed of multiple distinct components, each of which has a different function and is subject to different types of arrhythmias, a true precision approach to antiarrhythmic medication delivery would require the ability to target each subcomponent. As such, here we have uncovered and validated a suite of extracellular markers that can potentially be used for additional targeting efforts (e.g., targeted molecular ablation of the AVN in recalcitrant atrial fibrillation).

Our current work is limited by the need for clinical trials within humans to confirm the degree of resolution and signal penetration in larger, more dense hearts as well as the effect of CCS visualization on patient outcomes. Further, while CNTN2 (11) and NPTN (this work) have been validated in the human CCS, the other extracellular CCS markers described here will need to be verified in humans through the creation of functional human-specific antibodies and/or human scRNA-seq data of the CCS.

Overall, our study represents, for the first time to our knowledge, a proof of principle for antibody-based targeting of molecularly defined cardiac substructures in vivo and lays the groundwork for additional translational opportunities, including the targeting of alternative cargos such as contrast agents (MRI/CT), drugs (antiarrhythmics), and other therapeutics (RNA, DNA, small molecules) to the CCS and other cardiac substructures.

## Methods

### Mice.

Wild-type, CD1 mice were acquired from The Jackson Laboratory. Mice at indicated ages were used in accordance with the Institutional Animal Care and Use Committee of Stanford University. Both female and male mice were used for all experiment types described at a 1:1 ratio.

### Human CCS tissue.

Human CCS tissue (adult, *n =* 1, 65 years) was acquired from autopsies at the Stanford University Department of Pathology and appropriately deidentified. Tissues were fixed for 24 hours in 4% paraformaldehyde (Thermo Fisher Scientific, 50-980-487), washed in PBS for 10 minutes 3 times, and incubated in 30% sucrose in PBS for 24 hours at 4°C and then embedded in Tissue-Plus optimal cutting temperature (OCT) (Thermo Fisher Scientific, 23-730-571) for cryosection. Immunostaining was performed as detailed below.

### Bioinformatics analysis.

All bioinformatics analyses were performed on our preexisting scRNA-seq data set of the developing mouse CCS as previously described (NCBI/GEO database under accession number GSE132658) (16), with additional details in Supplemental Methods. Significance is presented as an adjusted *P* value that is based on Bonferroni’s correction using all features in the data set. SurfaceGenie, a web-based application, was used to predict candidate surface markers from the pool of significantly enriched genes (17). All putative cell surface markers were then confirmed manually using UniProt (18).

### Optical imaging agents.

Described antibody-dye conjugates consist of commercially acquired anti-Cntn2 goat polyclonal antibody (AF4439) and anti-NPTN goat polyclonal antibody (AF5360) (both R&D Systems) that have been covalently conjugated to a benign, NIR dye (IRDye800CW NHS ester; LI-COR Biosciences, 929-70020) using company specifications. Control agents (IgG-800) consisted of nonspecific IgG (Thermo Fisher Scientific, 50270683) conjugated to the same NIR dye. Unconjugated free dye was eliminated using Zeba Spin Desalting Columns (Pierce, 89891).

### Human anti-CNTN2 monoclonal Fab creation.

A phage display system was used (Promab Biotech, Inc) to screen for monoclonal Fab antibodies targeting the human CNTN2-His recombinant protein (Acro Biosystems, CN2-H5226). See Supplemental Methods for details.

### Monoclonal Fab in vivo targeting of the CCS.

The monoclonal anti-CNTN2 Fab was conjugated to the same, aforementioned NIR dye (IRDye800CW NHS ester, LI-COR, 929-70020) using company specifications. For conjugation to saporin, the Fab was first biotinylated using the Fluoreporter Mini-Biotin-XX Protein Labeling Kit (Invitrogen, F6347) according to company specifications. The biotinylated Fab (100 μg) was then added to streptavidin-conjugated saporin (25 μg) (Advanced Targeting Systems, IT-27) at room temperature prior to injection into wild-type CD1 mice. Control mice were injected with an equivalent amount of nonspecific human IgG conjugated to saporin (biotinylated human IgG and streptavidin-saporin) (Advanced Targeting Systems, IT-77). Surface ECGs were taken prior to injection and daily under inhaled sedation until euthanasia after 48 hours. Following euthanasia, the heart was then harvested and fixed in 4% paraformaldehyde for 24 hours prior to washing in PBS for 10 minutes 3 times. Hearts were then embedded in OCT, sectioned, and immunostained as detailed below.

### Delivery of optical imaging agents, surface ECG, and imaging.

Adult CD1 mice received systemic injections of either mNptn-800 or mCntn2-800 at indicated doses, by tail vein injection under inhaled sedation. Controls consisted of mice injected with nonspecific IgG conjugated to the same NIR dye (IgG-800). Surface ECGs were taken prior to injection and daily under inhaled sedation until euthanasia after 24, 48, or 72 hours as indicated. Following euthanasia, the heart, along with all other major organs were then harvested and imaged using closed-field (Pearl Impulse, LI-COR) fluorescence imaging. Closed-field fluorescence images were analyzed with ImageStudio (LI-COR) by calculating mean fluorescence intensity (MFI) within a tailored region of interest (ROI). The ROI was hand drawn around the SAN tissue to quantify conduction tissue MFI. To assess background MFI, an ROI was created on the left atrial appendage. The conduction-to-background MFI ratio (signal to background ratio, SBR) was assessed for each mouse to evaluate the temporal effect on the fluorescence contrast produced by each agent. Live imaging was performed using the FLARE Intraoperative NIR Fluorescence Imaging System (15).

### Immunofluorescence.

Immunofluorescence staining was carried out by following a previous protocol with minor modifications (34). Briefly, all tissue samples were washed in PBS prior to fixation overnight in 4% paraformaldehyde (Thermo Fisher Scientific, 50-980-487) at 4°C. Hearts were then washed in PBS for 15 minutes 3 times prior to incubation in 30% sucrose in PBS overnight at 4°C and then embedded in Tissue-Plus OCT (Thermo Fisher Scientific, 23-730-571). Tissues were cut as cryosections of 10 μm thickness and stored at –80°C. The sections were dried for 1 hour prior to use, rehydrated in PBS, washed 3 times in PBST (PBS + 0.1% Triton X-100) and then blocked (PBST + 0.5% bovine serum albumin) for 1 hour at room temperature. Following this, the sections were incubated with primary antibodies diluted in blocking solution overnight at 4°C in humid chambers. On the second day, after washing 3 times with PBST, the sections were incubated with secondary antibody for 2 hours at room temperature. After additional washing with PBS for 5 minutes 3 times, the sections were mounted with mounting media containing DAPI (Vector Laboratories, H-1200). All primary and secondary antibodies used are detailed in Supplemental Methods. Images were taken with a Zeiss Axio Imager microscope or Zeiss LSM980 inverted confocal microscope at the Neuroscience Microscope Service (NMS) facility at Stanford University. Negative controls for immunostaining included the use of primary or secondary antibodies alone. A minimum of 4 biological (different hearts) and 4 technical (different slides/heart) replicates were used for each antibody staining.

### RNAscope in situ hybridization.

RNAscope Multiplex Fluorescent v2 (catalog 323100) was used per manufacturer’s suggested protocol. The following murine probes were used: Mm-Cpne5-C3 (catalog 496711-C3), Mm-Hcn4-C2 (catalog 421271-C2), Mm-Ntm-C1 (catalog 489111), Mm-Pcdh17-C2 (catalog 489901-C2), Mm-Slc22a1-C1 (catalog 532931), Mm-Slit2-C1 (catalog 449691), and Mm-Slitrk5-C1 (catalog 451891). All images were taken with a Zeiss Axio Imager microscope at the NMS facility at Stanford University. A minimum of 3 biological (different hearts) and 4 technical (different slides/heart) replicates were used for each in situ hybridization.

### iDISCO+.

For a detailed protocol, please see https://idisco.info/idisco-protocol/ Hearts acquired from mice systemically injected with mCntn2-800 were fixed and optically cleared per protocol. As the fluorescent probe mCntn2-800 was injected intravenously already prior to fixation, no primary or secondary antibodies were applied. Permeabilization was deferred given the lack of need for incubation with additional antibodies. At least 1 day after clearing, iDISCO+ samples were imaged on a light-sheet microscope (Ultramicroscope II, LaVision Biotec) at the NMS facility at Stanford University. A minimum of 4 biological (hearts from separately injected mice) replicates were used for each optical clearing.

### Statistics.

Two-tailed, unpaired Student’s *t* test was used to evaluate differences between groups in all cases. For multiple comparison analyses, 1- or 2-way ANOVA with Tukey’s post hoc test was employed as listed. Data are represented as mean ± SD. A *P* value of less than 0.05 was considered statistically significant. Sample sizes are detailed within figure legends and text. For single-cell analyses, significance is presented as an adjusted *P* value based on Bonferroni’s correction using all features in the data set.

### Study approval.

All animal studies were reviewed and approved in accordance with the Institutional Animal Care and Use Committee of Stanford University, Stanford, California. Human cardiac tissue samples for immunofluorescence staining were acquired from the Stanford University Department of Pathology, Stanford and, as deidentified cadaveric samples, exempt from human subject review.

## Author contributions

WRG and SMW conceived the study. NSVB, JWB, SM, FXG, SL, NP, DPC, CK, and ELR provided significant input on experimental design. WRG, BMB, and LD performed all described wet experiments in collaboration with NSVB (image acquisition/analysis), SM and DS (immunofluorescence/pathology), ERR and SR (murine studies), FXG and SL (scRNA-seq analysis), and DPC (antibody production and conjugation). WRG and SMW provided financial support for the studies. WRG and SMW wrote the manuscript.

## Supplementary Material

Supplemental data

Supplemental tables 1-6

Supplemental video 1

Supplemental video 2

Supplemental video 3

Supplemental video 4

## Version 1 08/11/2022

In-Press Preview

## Version 2 10/17/2022

Electronic publication
