# Supplementary material for: In vivo visualization and molecular targeting of the cardiac conduction system
Source: J Clin Invest. 2022 Oct 17;132(20):e156955. doi: 10.1172/JCI156955 (PMC9566899; doi:10.1172/JCI156955)
Supplement: Supplemental data [file jci-132-156955-s008.pdf]

Supplemental Figure Legends:

Supplemental Figure 1. Cntn2-800 Dosage and Timecourse Analyses. (**A**) Experimental workflow for dosage analysis. Whole hearts from a wild-type (WT) mouse injected 3 days prior with polyclonal Cntn2-800 or non-specific IgG-800 (Control) at listed dosages. n = sample size per dose. Representative heart images with near infrared (NIR) signal demonstrating labeling of the CCS (Blue->Red = Lowest->Highest signal). (**B**) Bar graph showing a significant increase in mean signal to background ratio (SBR) at each dosage as compared to control IgG SBR using a one-way ANOVA with Tukey's post-hoc test. Significance considered at $p<0.05$. (**C**) Workflow of timecourse analysis. Each WT mouse received a single 75ug injection of Cntn2-800 and hearts were harvested at Day 1, 2, 3 or 4 for NIR imaging. n = 3 mice per timepoint. (**D**) Bar graph showing no significant change in mean SBR even out to 4 days post-injection to Day 1 following injection. Statistical analyses performed using a one-way ANOVA with Tukey's post-hoc test. Significance considered at $p<0.05$.

Supplemental Figure 2. Specific Labeling of the Murine CCS Following mCntn2-800 Systemic Injection. Mouse heart sections from mice injected with mCntn2-800 (n=3), demonstrating mCntn2-800 signal (cyan) labeling the (**A-B**) atrioventricular node (AVN), (**C-D**) His bundle (His) and left bundle branch (LBB), and (**E-H**) Purkinje fibers (PF), as costained with anti-Cntn2 (red) immunostaining. Staining with anti-Cx40 (purple) shows expected absence in AVN (**A-B**) and expression in the His and LBB (**C-D**). Anti-Cx43 (purple, **E-F**) labels the ventricular myocardium (VM) while anti-CD31 (purple, **G-H**) marks the vascular endothelium and not the mCntn2-800-positive CCS. DAPI (blue, nuclei). INT, internodal tract cells; IVS, interventricular septum.

Supplemental Figure 3. Human Anti-CNTN2 Monoclonal Fab Antibody Targets the CCS. (**A**) Titration ELISA with serial dilution of anti-CNTN2 Fab. 96-well plates were coated with recombinant human CNTN2 protein (2 ug/mL) and, following blocking, anti-CNTN2 Fab was added to the wells at various dilutions. Following the wash, anti-human Fab-HRP was added followed by TMB (3,3',5,5'-Tetramethylbenzidine) substrate for signal detection at OD450. (**B**) SDS-polyacrylamide gel electrophoresis of purified (left) and unpurified (right) Fab in reduced (~25kDa) and unreduced (~50kDa) states (i.e., ± 2.5% beta-mercaptoethanol). (**C**) Human Fab-dye conjugate (hCNTN2-800). (**D**) Wild-type mice (n=3) received a single tail-vein injection of hCNTN2-800 (150ug). After 1 day, hearts were harvested, fixed and sectioned for fluorescence staining. (**E**) Purkinje fibers (PF) labelled by hCNTN2-800 signal (red) and co-stained with Cx40 (green). DAPI (blue, nuclei). NIR, near-infrared.

Supplemental Figure 4. Anti-CNTN2-Saporin Conjugate Induces Targeted Cell Death of Major CCS Components. Immunofluorescence of the CCS (red, anti-Cntn2 immunostaining) showed subtotal loss of CCS cells within the sinoatrial node (SAN) and atrioventricular node (AVN) in wild-type mice injected 48 hours prior with a single tail-vein injection of either Control-Sap (100ug nonspecific human IgG similarly conjugated to Saporin) (n=6) or hCNTN2-Sap (100ug) (n=3). DAPI (blue, nuclei).

Supplemental Figure 5. Anti-CNTN2-Saporin Conjugate Does Not Result in Increased Cell Death Within the Brain. Coronal section of the cerebral cortex from wild-type mice (n=3) injected 48 hours prior with a single tail-vein injection of either (**A-C**) Control-Saporin (100ug nonspecific human IgG similarly conjugated to Saporin) or (**D-F**) hCNTN2-Saporin (100ug). Immunofluorescence of the brain with neuronal cell bodies labeled with anti-NeuN (red) illustrating only extremely rare cells undergoing cell death (arrows), labeled by activated caspase 3 (aCasp3, cyan). Progressive magnification of boxed field as noted. DAPI (blue, nuclei).

Supplemental Figure 6. Gfra2 Labels the Murine SAN, AVN and His Bundle. Wild-type mice (n=4) heart sections (embryonic day 18.5) demonstrating (**A-C**) anti-Gfra2 (cyan) and anti-Cntn2 (red) co-labeling of the sinoatrial node (SAN) as opposed to the right atrium (RA) stained with anti-Cx40 (purple). Gfra2 (cyan) also labels the atrioventricular node (AVN) (**D-F**) and His bundle (His) (**G-I**). Ventricular myocardium (VM) stained with anti-Cx43 (purple). DAPI (blue, nuclei).

Supplemental Figure 7. Gfra2 Also Labels a Small Subpopulation of Vimentin+ Cells Surrounding Large Vessels in the Heart. Wild-type mice (n=4) heart sections (embryonic day 18.5) demonstrating anti-Gfra2 (cyan) additionally labelling a rare subpopulation of cells surrounding large cardiac vessels that are negative for endothelial marker CD31 (red, **A-C**) and vascular smooth muscle marker alpha-SMA (purple, **D-F**) but co-labels with a subpopulation of vimentin positive cells (**G-I**). Ventricular myocardium (VM). DAPI (blue, nuclei).

Supplemental Figure 8. Epha4 Labels the Murine Ventricular Conduction System. Wild-type mice (n=4) heart sections (embryonic day 16.5) demonstrating (**A-C**) anti-Epha4 (cyan) and anti-Cntn2 (red) co-labeling of Purkinje fiber cells (PF) as opposed to (**D-F**) the ventricular myocardium (VM) stained with anti-Cx43 (red), or (**G-I**) vascular endothelium stained with anti-CD31 (red). DAPI (blue, nuclei).

Supplemental Figure 9. Novel Cell Surface Markers Label Distinct Components of the Murine CCS. Fluorescence RNA *in situ* hybridization (FISH) staining of wild-type murine, embryonic day 16.5 cardiac tissue sections (n=3 for each). Distinct CCS components shown including the sinoatrial node (SAN), atrioventricular node (AVN), His bundle (His), bundle branches (BB) and Purkinje fiber (PF) cells (each component outlined by a solid line) for each gene marker. DAPI (blue) in all images. (**A, D**) FISH targeting the known SAN marker Smoc2 or AVN/His/BB/PF marker Cpne5 (red punctae) as well as novel markers Pcdh17 (**A**) or Slc22a1 (**D**) (cyan punctae). (**B-C**) FISH targeting the known CCS marker Hcn4 (red punctae) and known markers Slitrk5 (**B**) or Slit2 (**C**) (cyan punctae) mRNA. cSAN, compact SAN; INT, internodal tract; IVS, interventricular septum; MV, mitral valve; LA, left atrial myocardium; LBB, left bundle branch; LV, left ventricle; Prox, proximal; RA, right atrial myocardium; RBB, right bundle branch; RBC, red blood cells; SAN Tz, SA nodal transitional cells; VM, ventricular myocardium.

Supplemental Figure 10. The Murine CCS is Co-Labeled by Nptn and Cntn2. Wild-type mice (n=3) heart sections (postnatal day 10) demonstrating anti-Nptn (cyan) and anti-Cntn2 (red) co-labeling of the His bundle (His) and proximal right and left bundle branches (RBB/LBB) (**A-F**). Ventricular myocardium (VM) stained with anti-Cx43 (purple, **A-D**) while the vascular endothelium is labeled with anti-CD31 (purple, **E-F**). DAPI (blue, nuclei).

Supplemental Figure 11. NPTN is Enriched in the CCS of the Adult Human Heart. Immunofluorescence staining of anti-NPTN (cyan) in cardiac tissue sections from a 65 year-old human heart showing (**A**) His bundle co-stained with anti-HCN4 (red); (**B**) showing magnified border with the interventricular septum (IVS). DAPI (blue, nuclei). (**C**) Purkinje fibers (PF) labeled with NPTN (cyan) and immunostaining for known PF marker CX40 (red). (**D**) NPTN+ PF and bordering IVS labeled by anti-CX43 (purple).

Supplemental Figure 12. ECG Intervals are Unchanged in Mice Injected with Nptn-800. Sedated surface electrocardiograms (ECGs) with measured intervals (in msec) including RR and QTc (Bazett's corrected QT interval) in wild-type mice prior to (Day 0 = baseline) and daily (Day 1, Day 2, Day 3 = post-injection) following a single tail vein injection of Nptn-800 (150ug) in each animal. N=3 for all time points. Intervals (mean in msec +/- STD) on a given day post-injection were compared to each mouse's pre-injection control baseline (Day 0) using a one-way ANOVA with Tukey's post-hoc test. Significance considered at $p<0.05$.

Supplemental Movie Legends:

Supplemental Movie 1. iDISCO+ of an Intact Heart Following mCntn2-800 Systemic Injection Reveals High Resolution Labeling of the Entire CCS. iDISCO+ cleared heart harvested from a wild-type (CD1) mouse injected two days prior with mCntn2-800 (75ug). Near-infrared (800nm) signal (gold) from mCntn2-800 labeling the entire CCS.

Supplemental Movie 2. Live Imaging of the Murine CCS Following mCntn2-800 Systemic Injection. Simulated surgical scenario in which a wild type mouse received a tail vein injection of mCntn2-800 and after 48 hours was sedated and received a sternotomy, right atriotomy and right ventriculotomy to expose the fibrillating heart for direct live imaging with a FLARE™ Intraoperative Near-Infrared (NIR) Fluorescence Imaging System. Left: Merge of regular color video with superimposed NIR (800nm) signal (green) from mCntn2-800. Right: NIR signal (green) alone. Structures as labeled. AVN, atrioventricular node; His, His bundle; IVS, interventricular system; RA, right atrium; RV, right ventricle; SAN, sinoatrial node; SVC, superior vena cava.

Supplemental Movie 3. Ex Vivo NIR Imaging of the Murine CCS (SAN/AVN/His) Following mCntn2-800 Systemic Injection. Heart immediately removed following euthanization of a mouse having previously received a systemic injection of mCntn2-800 48 hours prior (Figure 3) and imaged *ex vivo* while still fibrillating with a FLARE™ Intraoperative Near-Infrared (NIR) Fluorescence Imaging System. Left: Merge of regular color video with superimposed NIR (800nm) signal (green) from mCntn2-800. Right: NIR signal (green) alone. Structures as labeled. AVN, atrioventricular node; His, His bundle; RA, right atrium; RV, right ventricle; SAN, sinoatrial node; SVC, superior vena cava.

Supplemental Movie 4. Ex Vivo NIR Imaging of the Murine CCS (PFs) Following mCntn2-800 Systemic Injection. Heart immediately removed following euthanization of a mouse having previously received a systemic injection of mCntn2-800 48 hours prior (Figure 3) and imaged *ex vivo* while still fibrillating with a FLARE™ Intraoperative Near-Infrared (NIR) Fluorescence Imaging System. Left: Merge of regular color video with superimposed NIR (800nm) signal (green) from mCntn2-800. Right: NIR signal (green) alone. Structures as labeled. LV, left ventricle; PFs, Purkinje fibers; RV, right ventricle.

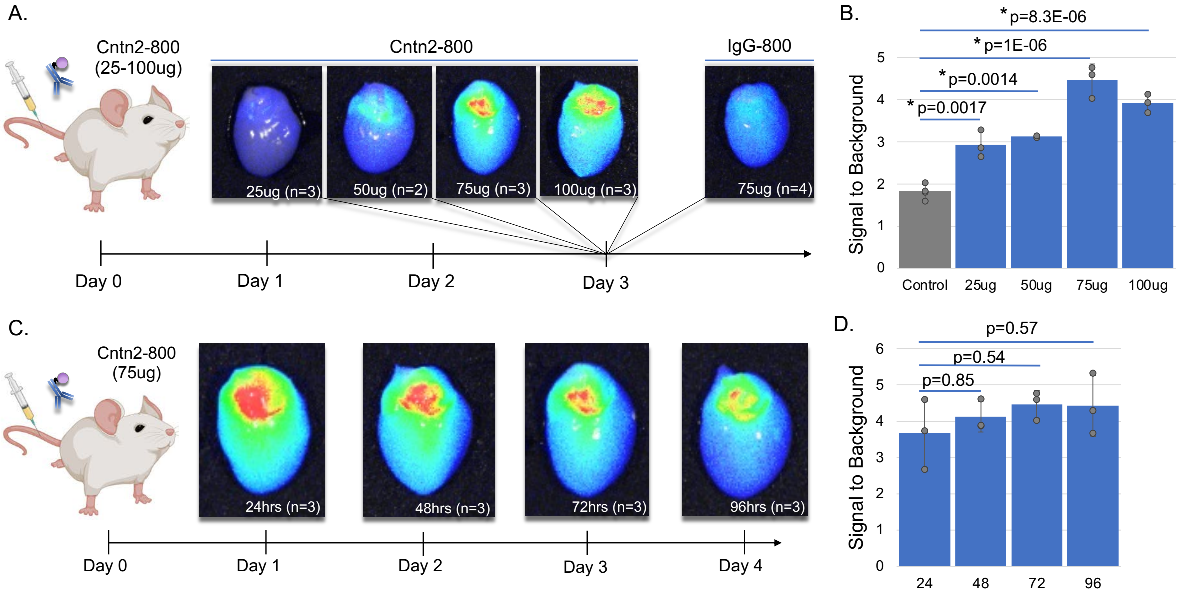

A.
Cntn2-800
(25-100ug)
Cntn2-800
IgG-800
25ug (n=3)
50ug (n=2)
75ug (n=3)
100ug (n=3)
75ug (n=4)
Day 0
Day 1
Day 2
Day 3
B.
*p=8.3E-06
*p=1E-06
*p=0.0014
*p=0.0017
Signal to Background
0
1
2
3
4
5
Control
25ug
50ug
75ug
100ug
C.
Cntn2-800
(75ug)
24hrs (n=3)
48hrs (n=3)
72hrs (n=3)
96hrs (n=3)
Day 0
Day 1
Day 2
Day 3
Day 4
D.
p=0.57
p=0.54
p=0.85
Signal to Background
0
1
2
3
4
5
6
24
48
72
96

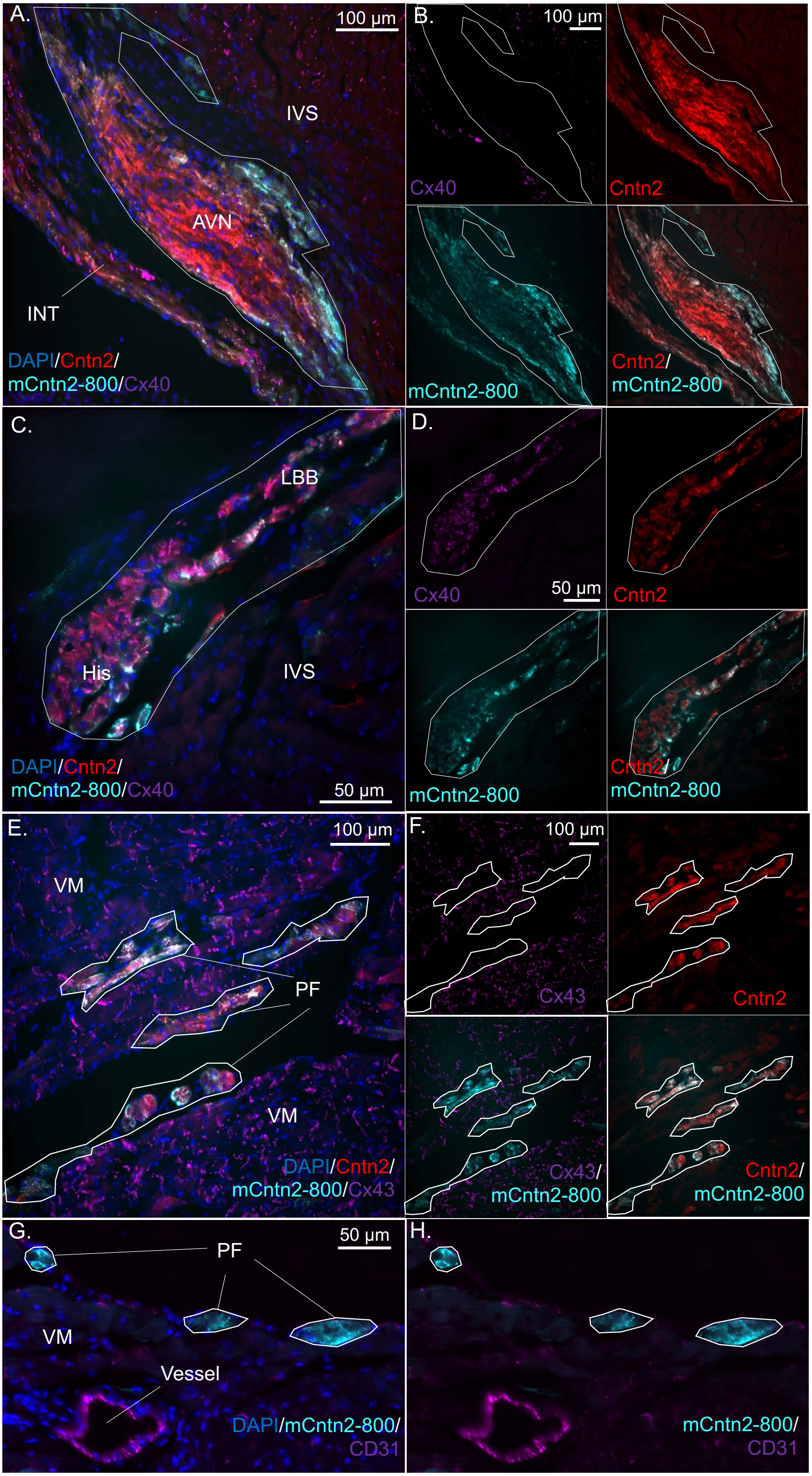
A.
100 µm
IVS
AVN
INT
DAPI/Cntn2/
mCntn2-800/Cx40
B.
100 µm
Cx40
Cntn2
mCntn2-800
Cntn2/
mCntn2-800
C.
LBB
His
IVS
DAPI/Cntn2/
mCntn2-800/Cx40
50 µm
D.
Cx40
50 µm
Cntn2
mCntn2-800
Cntn2/
mCntn2-800
E.
100 µm
VM
PF
VM
DAPI/Cntn2/
mCntn2-800/Cx43
F.
100 µm
Cx43
Cntn2
Cx43/
mCntn2-800
Cntn2/
mCntn2-800
G.
50 µm
PF
VM
Vessel
DAPI/mCntn2-800/
CD31
H.
mCntn2-800/
CD31

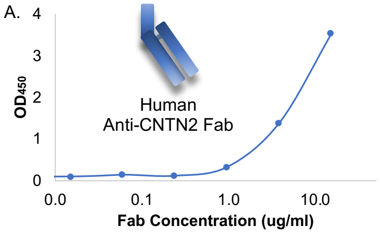

Fab Dilution (fold)	OD450	Fab Concentration (ug/ml)
10	3.5315	15
40	1.383	3.75
160	0.322	0.938
640	0.1125	0.234
2560	0.138	0.059
10240	0.0955	0.015

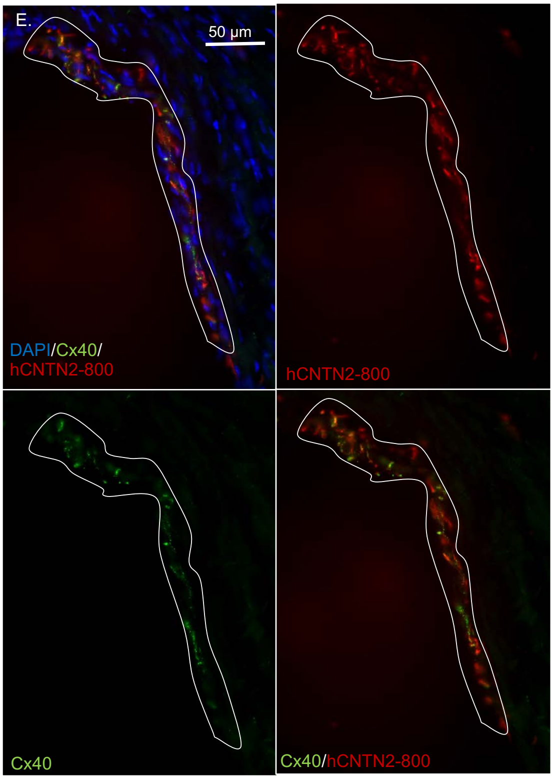

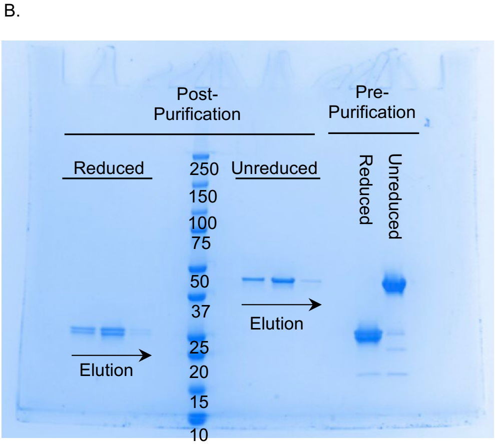

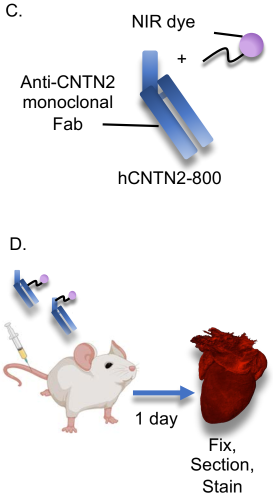

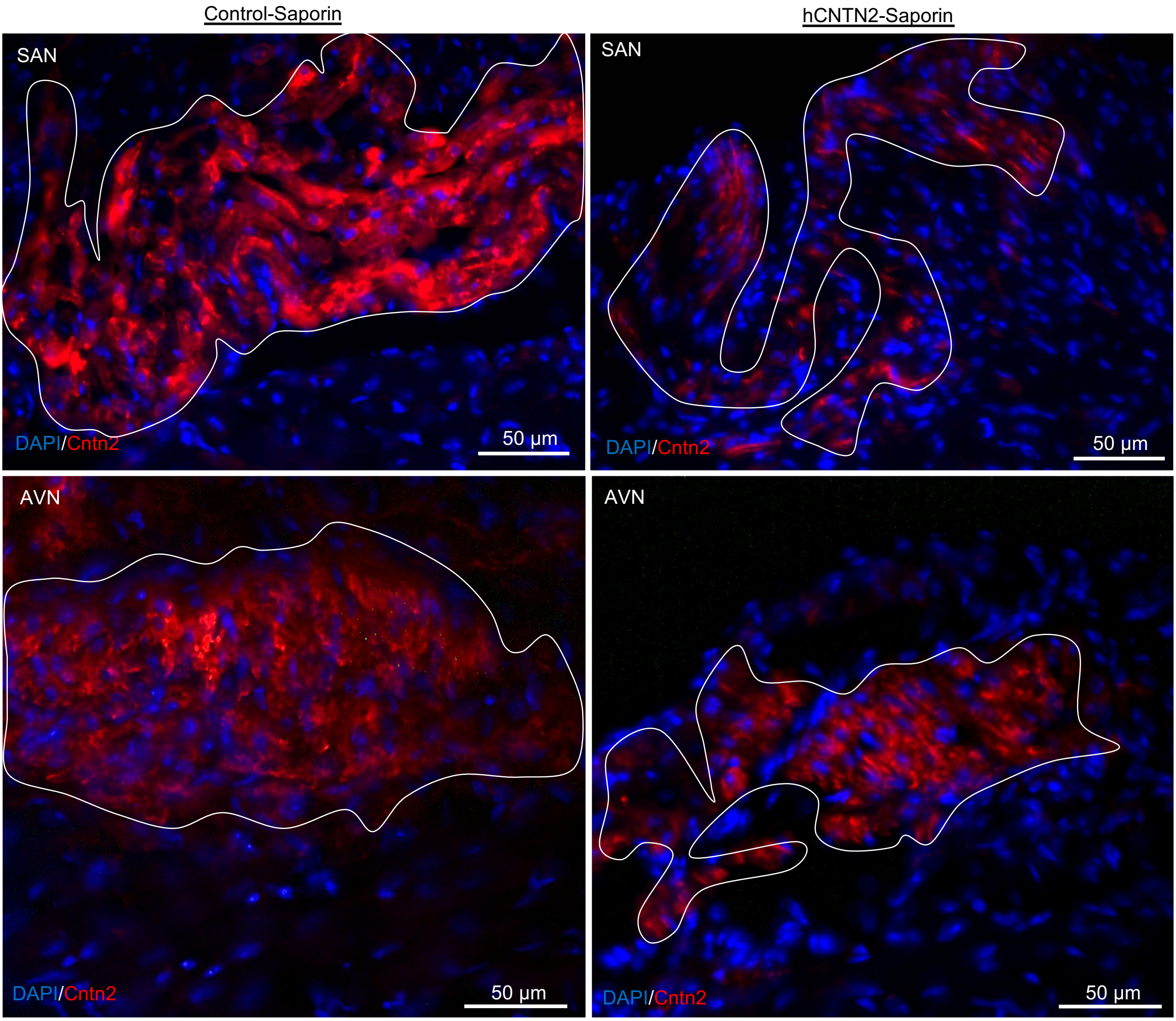

Control-Saporin
hCNTN2-Saporin
SAN
SAN
AVN
AVN
DAPI/Cntn2
DAPI/Cntn2
DAPI/Cntn2
DAPI/Cntn2
50 μm
50 μm
50 μm
50 μm

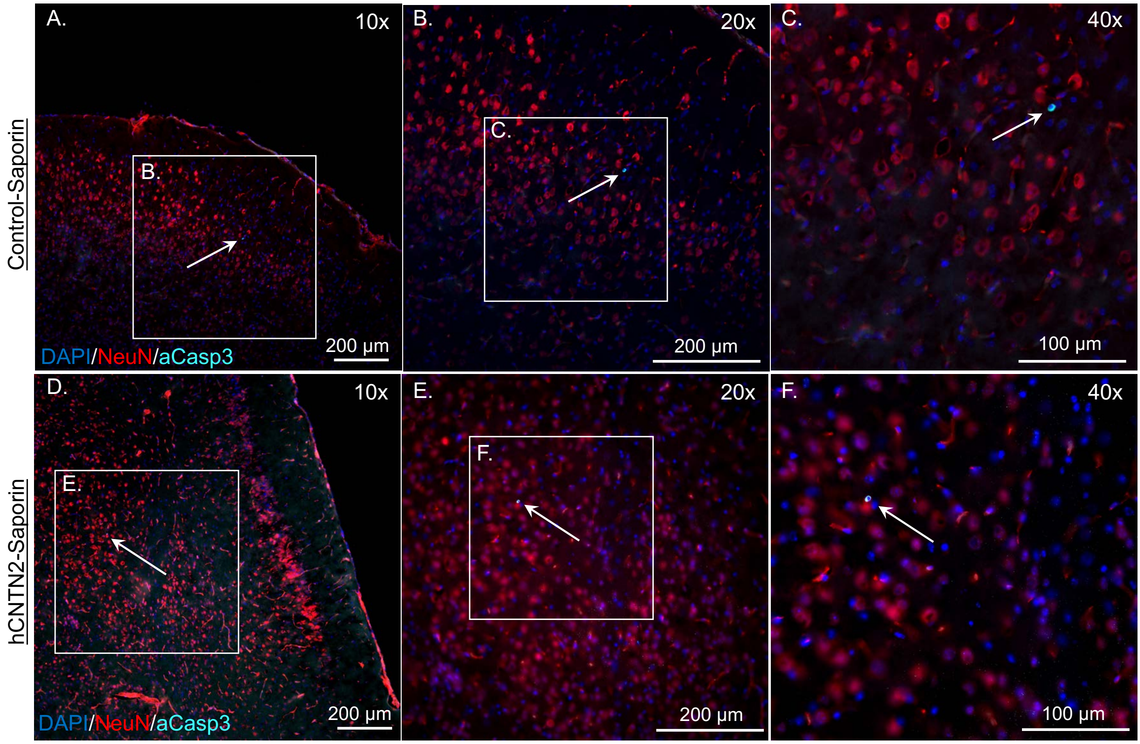

Control-Saporin
hCNTN2-Saporin
A.
10x
B.
20x
C.
40x
D.
10x
E.
20x
F.
40x
DAPI/NeuN/aCasp3
DAPI/NeuN/aCasp3
200 μm
200 μm
100 μm
200 μm
200 μm
100 μm

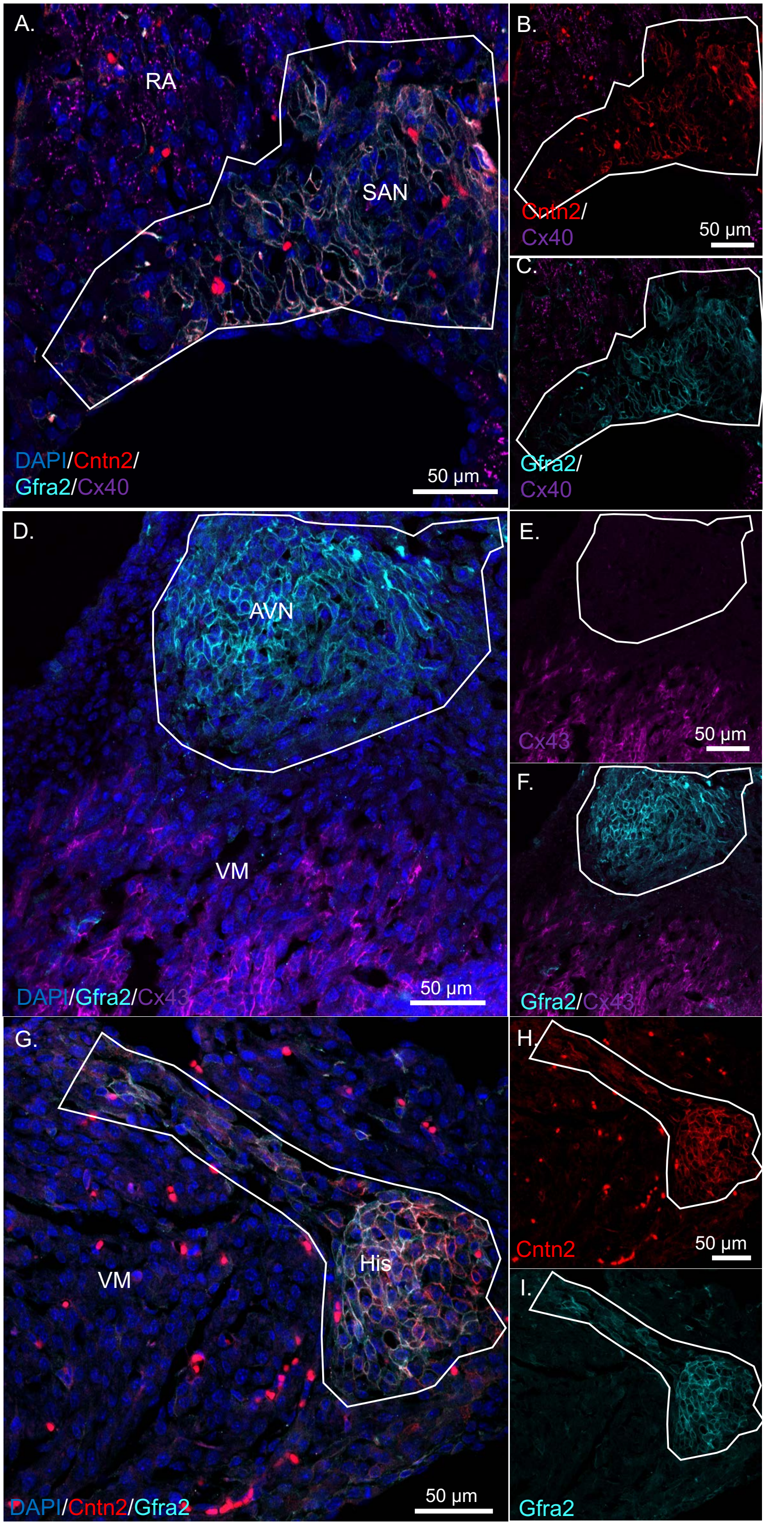
A.
RA
SAN
DAPI/Cntn2/
Gfra2/Cx40
50 µm
B.
Cntn2/
Cx40
50 µm
C.
Gfra2/
Cx40
D.
AVN
VM
DAPI/Gfra2/Cx43
50 µm
E.
Cx43
50 µm
F.
Gfra2/Cx43
G.
VM
His
DAPI/Cntn2/Gfra2
50 µm
H.
Cntn2
50 µm
I.
Gfra2

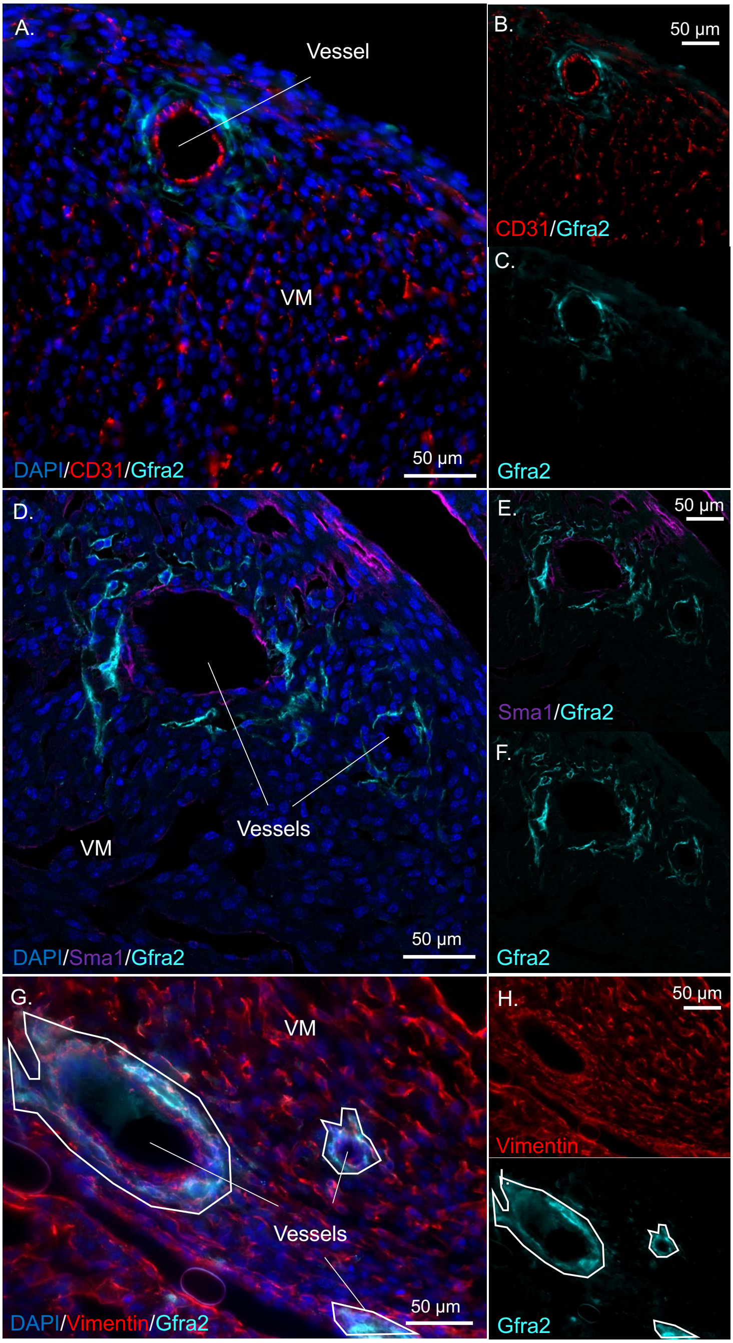

A.
Vessel
VM
DAPI/CD31/Gfra2
50 µm
B.
50 µm
CD31/Gfra2
C.
Gfra2
D.
Vessels
VM
50 µm
DAPI/Sma1/Gfra2
E.
50 µm
Sma1/Gfra2
F.
Gfra2
G.
VM
Vessels
50 µm
DAPI/Vimentin/Gfra2
H.
50 µm
Vimentin
I.
Gfra2

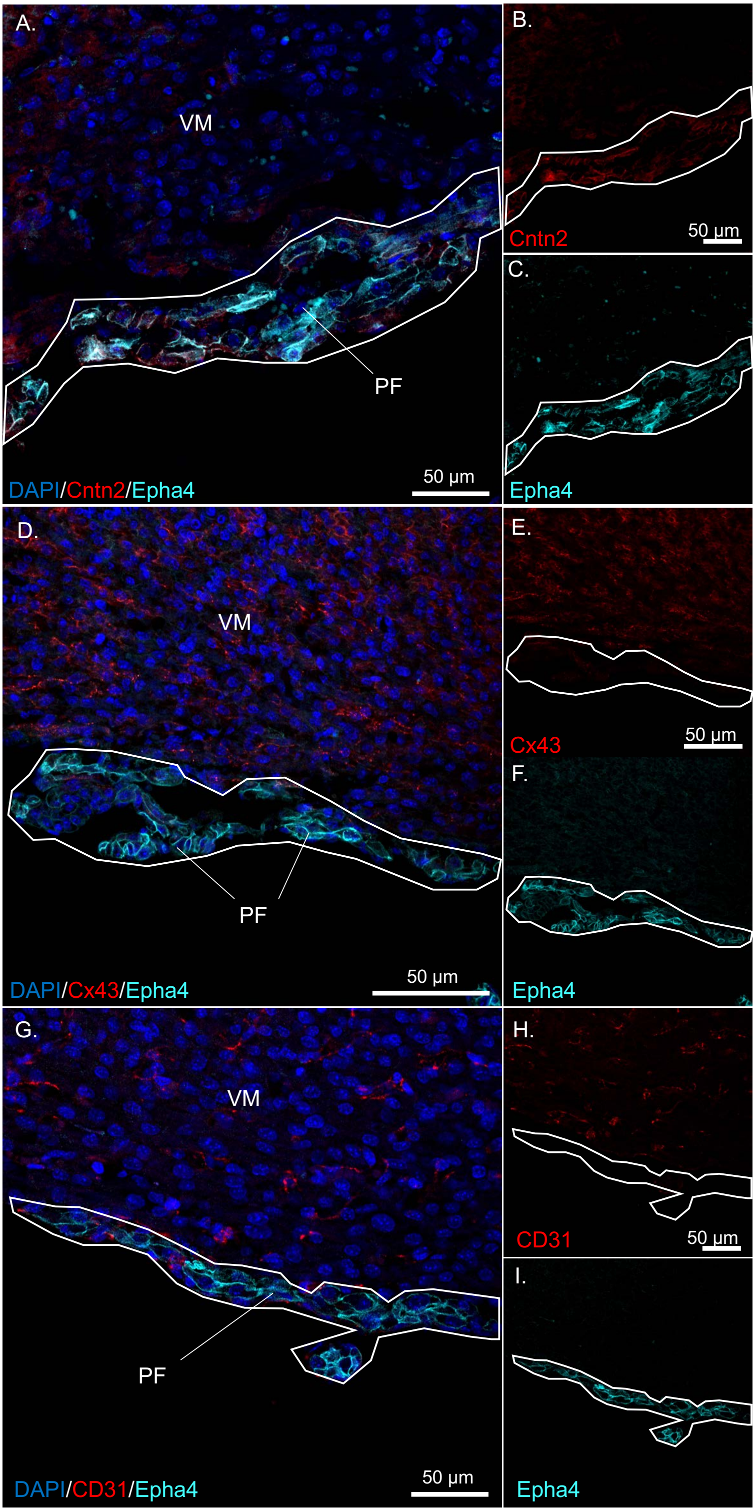
A.
VM
PF
DAPI/Cntn2/Epha4
50 µm
B.
Cntn2
50 µm
C.
Epha4
D.
VM
PF
DAPI/Cx43/Epha4
50 µm
E.
Cx43
50 µm
F.
Epha4
G.
VM
PF
DAPI/CD31/Epha4
50 µm
H.
CD31
50 µm
I.
Epha4

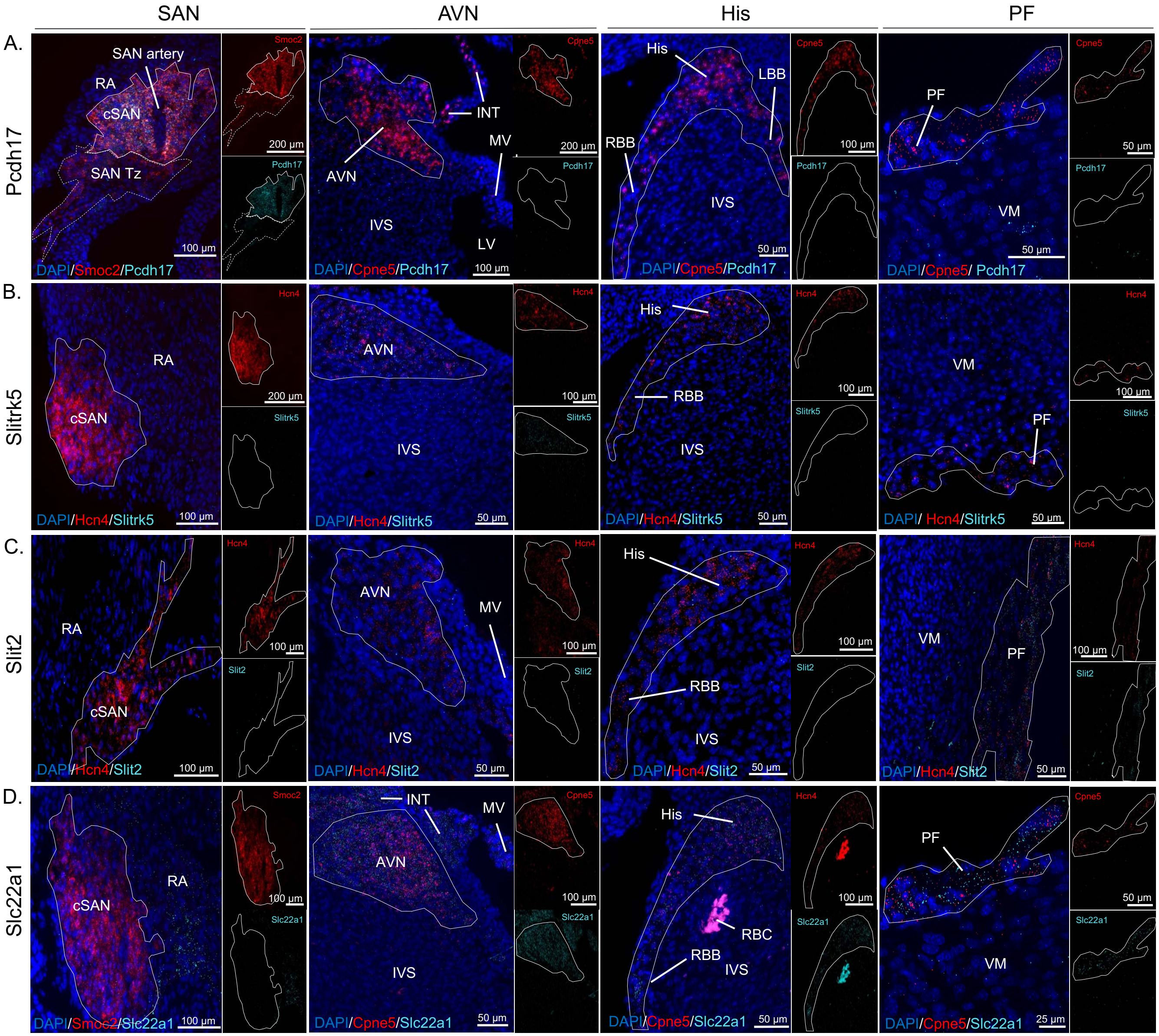

SAN
AVN
His
PF
A.
Pcdh17
SAN artery
RA
cSAN
SAN Tz
Smoc2
Pcdh17
200 µm
100 µm
DAPI/Smoc2/Pcdh17
INT
AVN
MV
IVS
LV
Cpne5
DAPI/Cpne5/Pcdh17
His
LBB
RBB
IVS
50 µm
PF
VM
DAPI/Cpne5/ Pcdh17
B.
Slitrk5
RA
cSAN
Hcn4
Slitrk5
DAPI/Hcn4/Slitrk5
AVN
IVS
His
RBB
VM
PF
C.
Slit2
RA
cSAN
Hcn4
Slit2
DAPI/Hcn4/Slit2
AVN
MV
IVS
His
RBB
VM
PF
D.
Slc22a1
cSAN
RA
Smoc2
Slc22a1
DAPI/Smoc2/Slc22a1
INT
MV
AVN
IVS
Cpne5
DAPI/Cpne5/Slc22a1
His
RBC
RBB
IVS
Hcn4
PF
VM
25 µm

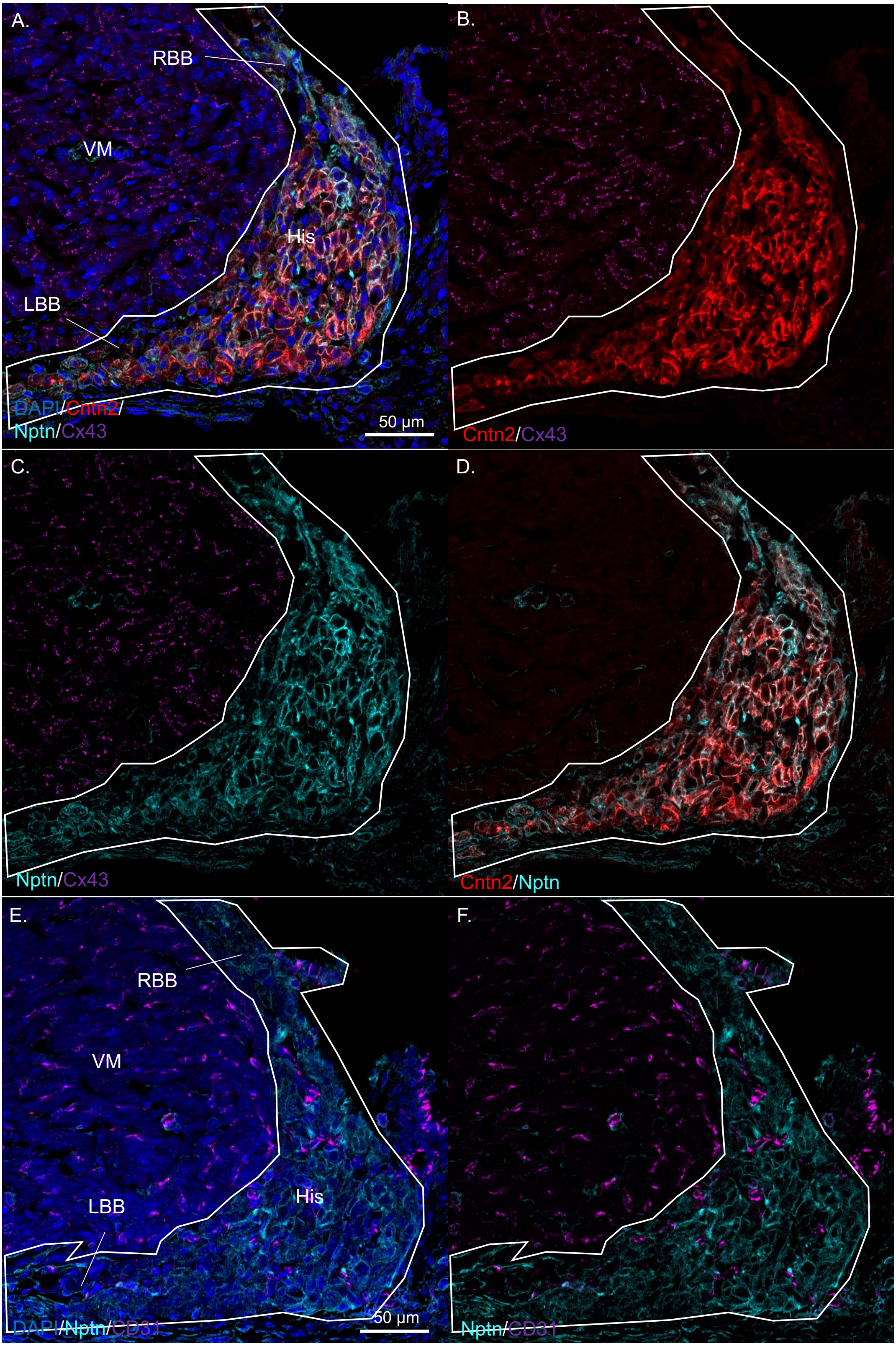
A.
RBB
VM
His
LBB
DAPI/Cntn2/
Nptn/Cx43
50 µm
B.
Cntn2/Cx43
C.
Nptn/Cx43
D.
Cntn2/Nptn
E.
RBB
VM
His
LBB
DAPI/Nptn/CD31
50 µm
F.
Nptn/CD31

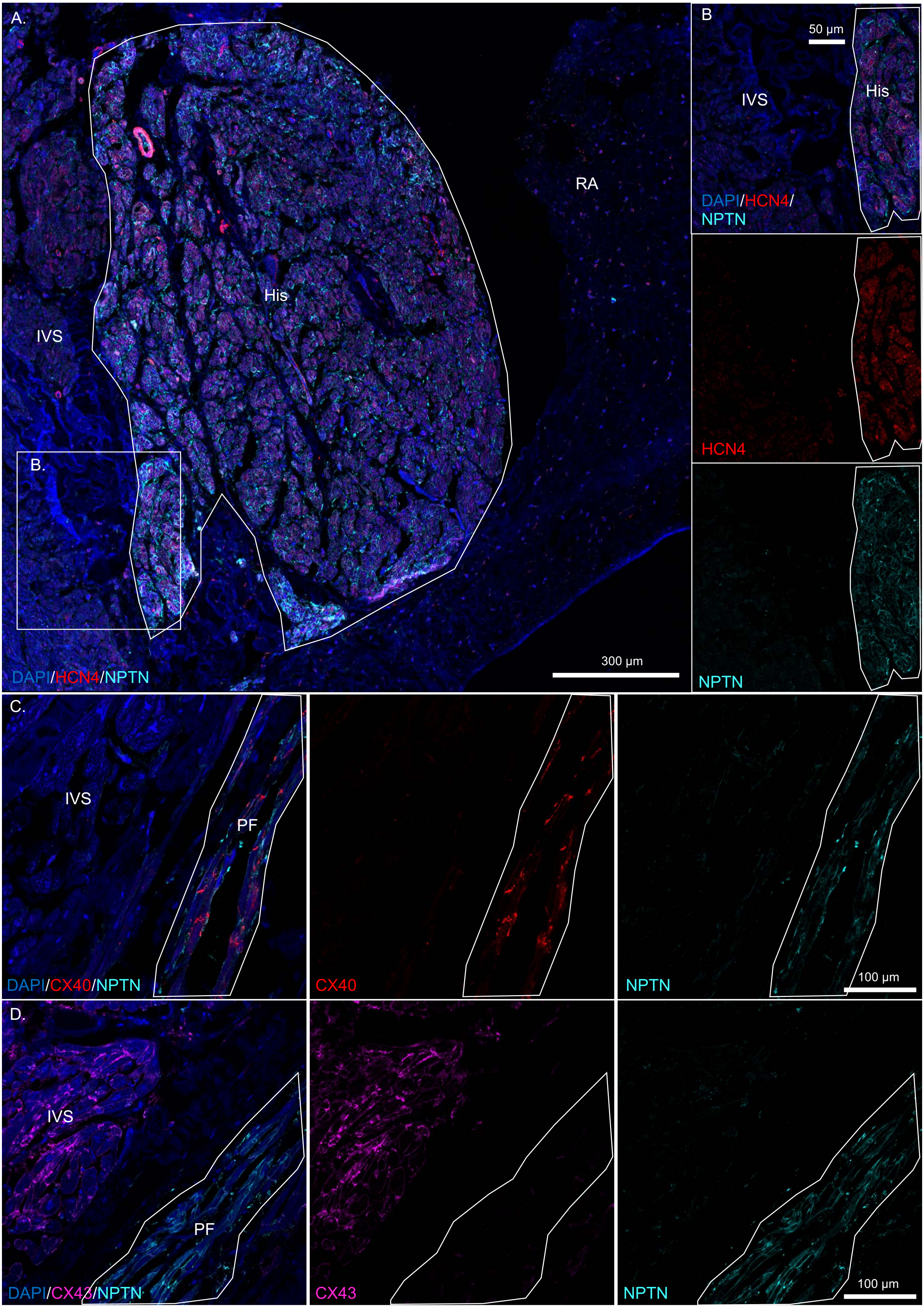
A.
His
RA
IVS
B.
DAPI/HCN4/NPTN
300 µm
B
50 µm
IVS
His
DAPI/HCN4/
NPTN
HCN4
NPTN
C.
IVS
PF
DAPI/CX40/NPTN
CX40
NPTN
100 µm
D.
IVS
PF
DAPI/CX43/NPTN
CX43
NPTN
100 µm

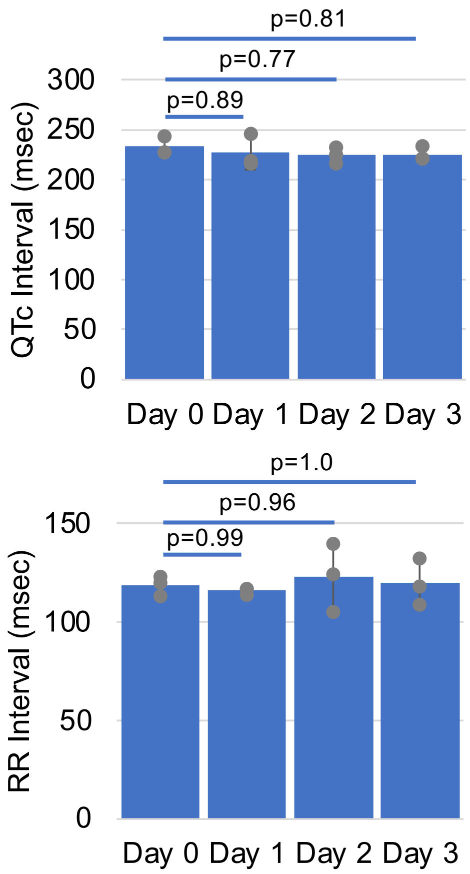
p=0.81
p=0.77
p=0.89
QTc Interval (msec)
300
250
200
150
100
50
0
Day 0 Day 1 Day 2 Day 3
p=1.0
p=0.96
p=0.99
RR Interval (msec)
150
100
50
0
Day 0 Day 1 Day 2 Day 3

SUPPLEMENTAL METHODS

Mice: Wild-type, CD1 mice were acquired from Jackson Laboratory (Sacramento, CA). Mice at indicated ages were used in accordance with the Institutional Animal Care and Use Committee of Stanford University. Both female and male mice were used for all experiment types described at a 1:1 ratio. For systemic injection of novel optical imaging tools, adult male and female mice were used at the age of 4-6 weeks. For immunofluorescence or FISH analyses, mice were used at indicated ages.

Human CCS Tissue: Human cardiac conduction system tissue (Adult, n=1, 65 years) with post-mortem intervals (PMI) less than 24 hours was acquired from autopsies at the Stanford University Department of Pathology and appropriately de-identified. Heart was fixed for 24 hours in 4% paraformaldehyde (Fisher, 50-980-487) and washed in PBS for 10 minutes three times (Thermofisher, 10010023). Conduction components including the SAN region (SVC-RA junction), AVN region (triangle of Koch) and PF region (free wall and IVS) were manually dissected and incubated in 30% sucrose in PBS for 24 hours at 4°C and then embedded in Tissue-Plus OCT (Fisher, 23-730-571). Tissue sections were cut as cryosections of 12 µm thickness and stored at -80°C. Immunostaining as detailed below.

Bioinformatics Analysis: All bioinformatics analyses were performed on our pre-existing single-cell RNA sequencing (scRNAseq) dataset of the developing mouse CCS (NCBI/GEO database under accession number GEO: GSE132658) (16). The Droplet platform data was de-multiplexed and mapped to mouse genome MM10 using CellRanger from 10x Genomics with default parameters. Cell filter, data normalization, and unsupervised analysis were carried out in Seurat version 2 per their recommended steps (35, 36). Significance is presented as an "adjusted p-value", which is based on the Bonferroni correction using all features in the dataset. Briefly, the cells were filtered by their gene number and UMI number. The threshold we used for gene number is 500 to 60,000, and UMI number is 1,000 to 5 million. Next, we used the LogNormalize function to normalize gene expression in each cell. Specifically, we calculated the expression value of genes by following this formula: log {(each gene expression level / total gene expression value)*10,000}. Average log fold change (avg_log FC) described in all data provided represents the log fold-change of the average expression between the two groups. To remove the unwanted sources of variations, we scaled the data with the "vars.to.regress" parameter based on the number of UMIs, percentage of ribosome genes, and Rn45s expression value. Furthermore, we found all the variable genes and used them to perform principal component analysis (PCA). Within all the PCs, we used the top 10 PCs to do clustering and tSNE analysis. In the tSNE analysis, we set the seed.use as 10 and perplexity as 30. Finally, we used the FindAllMarker function to identify the genes differentially expressing in the cell clusters. To be detected, the genes have to express in at least 25% of cells in one of the two comparing clusters and the differential expression level also should be higher than 25%. SurfaceGenie, a web-based application, was used to predict candidate surface marker from the pool of significantly enriched genes (17). All putative cell surface markers were then confirmed manually using UniProt (18).

Description of Optical Imaging Agents:
The generated optical imaging agents consist of commercially acquired antibodies (1. anti-Cntn2 Goat Polyclonal antibody - AF4439; and 2. anti-NPTN Goat Polyclonal antibody - AF5360) that have been covalently conjugated to a benign, near-infrared (NIR) dye (IRDye800CW NHS ester, LI-COR #929-70020) using company specifications. IRDye800CW is a NIR imaging probe with broad absorption (778 nm) and emission (794 nm) peaks that is nontoxic to rodents (37) and currently used in human clinical imaging trials (7). Control agents (IgG-800) consisted of non-specific IgG (Fisher, 50270683). In brief, 100 ug of polyclonal antibody (diluted in 100 uL PBS) was pH adjusted to a pH of 8.5 by adding 10 uL of 1.0 M potassium phosphate buffer (K_2HPO_4; pH 9). Next, one vial of IRDye800CW NHS ester was dissolved with 25 uL of ddH_2O. Dye and antibody were immediately mixed to achieve a dye/protein ratio of 1.5-2:1 and kept at RT in the dark for 2 hours. After 2 hours of incubation, the antibody-IRDye800CW conjugation mixture was put onto PBS-equilibrated Zeba™ Spin Desalting Columns (pH 7.4) (Pierce, #89891) and centrifuged at 1,500xg for 2 min to separate conjugate from free dye. Conjugated agents were then stored at 4°C, in the dark.

Human anti-CNTN2 Monoclonal Fab Creation:
A phage display system was used (Promab Biotech, Inc) to screen for monoclonal Fab antibodies targeting the human CNTN2-His recombinant protein (Acro Biosystems, #CN2-H5226). Please refer to Promab Biotechnologies (https://www.promab.com) for specific protocol details regarding phage display Fab screening, expression, purification and screening by ELISA.

Monoclonal Fab In Vivo Targeting of the CCS:
The monoclonal anti-CNTN2 Fab was conjugated to the same, aforementioned near-infrared (NIR) dye (IRDye800CW NHS ester, LI-COR, #929-70020) using company specifications. For conjugation to Saporin, the Fab was first biotinylated using the fluoreporter mini-biotin-XX protein labeling kit (Invitrogen, #F6347) according to company specifications. The biotinylated-Fab (100ug) was then added to streptavidin-conjugated Saporin (25ug) (ATS, #IT-27) at room temperature prior to injection into each wild-type CD1 mouse. Six biological replicates were performed and compared to six control CD1 mice injected with an equivalent amount of random human IgG conjugated to Saporin (biotinylated human IgG and streptavidin-Saporin) (ATS, #IT-77). Surface electrocardiograms were taken prior to injection and daily under inhaled sedation until euthanasia after 48 hours. Following euthanasia, the heart was then harvested, fixed in 4% paraformaldehyde for 24hours prior to washing three times 10 minutes in 1x PBS. Hearts were then embedded in OCT, sectioned and stained as detailed in immunofluorescence methods.

Delivery of Optical Imaging Agents, Surface ECG and Imaging:
For systemic application, adult, CD1 mice were administered of either mNptn-800 (150ug) or mCntn2-800 (75ug), diluted in 100 ul of sterile PBS (Thermofisher, 10010023), by tail-vein injection under inhaled sedation (isoflurane 3.5%). Controls

consisted of mice injected with IgG-800 (75ug). Surface electrocardiograms were taken prior to injection and daily under inhaled sedation until euthanasia after 24, 48 or 72 hours as indicated. Following euthanasia, the heart, along with all other major organs were then harvested and imaged using closed-field (Pearl Impulse, LI-COR, Lincoln, NE) fluorescence imaging (FLI). Subsequently, each heart was processed for immunofluorescence or iDISCO+ as detailed below.

Closed-field fluorescence images were analyzed with ImageStudio (LI-COR) by calculating mean fluorescence intensity (MFI) within a tailored region of interest (ROI). The ROI was hand drawn around the sinoatrial nodal (SAN) tissue to quantify conduction tissue MFI. To assess background MFI, an ROI was created on the left atrial appendage (LAA). The conduction-to-background MFI ratio (Signal to Background Ratio or "SBR") was assessed for each mouse to evaluate the temporal effect on the fluorescence contrast produced by each agent. Live imaging was performed using the FLARE™ Intraoperative NIR Fluorescence Imaging System (15).

Immunofluorescence: Immunofluorescence staining was carried out by following a previous protocol with minor modifications.(34) Briefly, all tissue samples (including wild-type CD1 mouse hearts or human tissue sections from indicated gestational ages as well as hearts from postnatal mice previously injected with optical imaging agents) were isolated by dissection, washed in PBS prior to fixation overnight in 4% paraformaldehyde (Fisher, 50-980-487) at 4°C. Hearts were then washed in PBS for 15 min three times prior to incubation in 30% sucrose in PBS overnight at 4°C and then embedded in Tissue-Plus OCT (Fisher, 23-730-571). Tissues were cut as cryosections of 10 µm thickness and stored at -80°C. The sections were dried for 1 hour prior to use, rehydrated in PBS, washed three times in PBST (PBS + 0.1% Triton X100) and then blocked (PBST + 0.5% Bovine serum albumin) for 1 hour at room temperature. Following this, the sections were incubated with primary antibodies diluted in blocking solution overnight at 4°C in humid chambers. Primary antibodies used included: Anti-human/rat/mouse Alpha-Smooth Muscle Actin Rabbit Monoclonal (Abcam, ab124964) at 1:75 dilution; Anti-human CD31 Rabbit Polyclonal (Abcam, ab28364) at 1:100 dilution; Anti-mouse Connexin 40 Rabbit Polyclonal (Alpha Diagnostics, Cx40-A) at 1:100 dilution; Anti-human Connexin-43 Rabbit Polyclonal (Sigma-Aldrich, C6219) at 1:100 dilution; Anti-mouse/human/rat Cntn2/TAG1 Goat Polyclonal (R&D, AF4439) at 1:100 dilution; Anti-mouse EphA4 Goat Polyclonal (R&D, AF641) at 1:100 dilution; Anti-mouse/human GFRA2 Goat Polyclonal (R&D, AF429) at 1:100 dilution; Anti-human/rat/mouse HCN4 Rabbit Polyclonal (Sigma-Aldrich, AB5808) at 1:50 dilution; Anti-mouse Hcn4 Rat Monoclonal [SHG 1E5] (Abcam, ab32675) at 1:75 dilution; Anti-human/mouse NeuN Mouse Monoclonal (Sigma-Aldrich, MAB377) at 1:200 dilution; Anti-mouse NPTN55 Sheep Polyclonal (R&D, AF7818) at 1:200 dilution; Anti-mouse/human NPTN65 Goat Polyclonal (R&D, AF5360) at 1:100 dilution; Anti-human/rat/mouse Vimentin Rabbit Monoclonal (Abcam, ab92547) at 1:100.

On the second day, after washing three times with PBST, the sections were incubated with secondary antibody for 2 hours at room temperature. The following secondaries were used at a 1:500 dilution: Donkey anti-goat IgG Alexa Fluor 555 (Invitrogen, A-

21432), Chicken anti-Rabbit IgG Alexa Fluor 488 (Invitrogen, A-21441), Donkey anti-Rabbit IgG Alexa Fluor 647 (Invitrogen, A-31573) and Chicken anti-Rat IgG AlexaFluor 488 (Invitrogen A-21470). After additional washing with PBS for 5 minutes three times, the sections were mounted with mounting media containing DAPI (Vector Laboratories, H-1200). All images were taken with a Zeiss Axioimager microscope or Zeiss LSM980 inverted confocal microscope at Stanford University. Negative controls for immunostaining included the use of primaries or secondary antibodies alone. A minimum of 4 biological (different hearts) and 4 technical (different slides/heart) replicates were used for each antibody staining.

RNAscope *in situ* hybridization: RNAscope® Multiplex Fluorescent v2 (Cat. #323100) was used per manufacturer suggested protocol. The following murine probes were used: Mm-Cpne5-C3 - Cat No. 496711-C3, Mm-Hcn4-C2 - 421271-C2, Mm-Ntm-C1 - Cat No. 489111, Mm-Pcdh17-C2 – Cat No. 489901-C2, Mm-Slc22a1-C1 – Cat No. 532931, Mm-Slit2-C1 – Cat No. 449691, and Mm-Slitrk5-C1 – Cat No. 451891. All images were taken with Axioimager microscope at Neuroscience Microscope Service (NMS) facility at Stanford University. A minimum of 3 biological (different hearts) and 4 technical (different slides/heart) replicates were used for each *in situ* hybridization.

iDISCO+: For detailed protocol, please see https://idisco.info/idisco-protocol/. Hearts acquired from mice systemically injected with mCntn2-800 were fixed and optically cleared per protocol. As the fluorescent probe mCntn2-800 was injected intravenously already prior to fixation, no primary or secondary antibodies were applied. Additionally the permeabilization step was deferred given the lack of need for incubation with additional exogenous antibodies. At least one day after clearing, iDISCO+ samples were imaged on a light sheet microscope (Ultramicroscope II, LaVision Biotec) equipped with a sCMOS camera (Andor Neo) and a 2x/0.5 NA objective lens (MVPLAPO 2x) equipped with a 6 mm working distance dipping cap. Version v285 of the Imspector Microscope controller software was used. We imaged using the 800-nm laser. The samples were scanned with a step-size of 3 μm using the continuous light-sheet scanning method. A minimum of 4 biological (hearts from separately injected mice) replicates were used for each optical clearing. Images and videos were processed using Imaris (Oxford Instruments) for 3D visualization.
